# PTPN23 binds the dynein adaptor BICD1 and is required for endocytic sorting of neurotrophin receptors

**DOI:** 10.1242/jcs.242412

**Published:** 2020-03-30

**Authors:** Marta I. Budzinska, David Villarroel-Campos, Matthew Golding, Anne Weston, Lucy Collinson, Ambrosius P. Snijders, Giampietro Schiavo

**Affiliations:** 1 Department of Neuromuscular Diseases, UCL Queen Square Institute of Neurology, University College London, London WC1N 3BG, UK; 2 William Harvey Research Institute, Queen Mary University of London, London EC1M 6BQ, UK; 3 Electron Microscopy, The Francis Crick Institute, 1 Midland Road, London NW1 1ST, UK; 4 Proteomics Science Technology Platforms, The Francis Crick Institute, 1 Midland Road, London NW1 1ST, UK; 5 UK Dementia Research Institute, University College London, London WC1E 6BT, UK; 6 Discoveries Centre for Regenerative and Precision Medicine, University College London Campus, London WC1N 3BG, UK

**Keywords:** Intracellular sorting, Motor neuron, p75^NTR^, Trafficking, TrkB

## Abstract

Signalling by target-derived neurotrophins is essential for the correct development of the nervous system and its maintenance throughout life. Several aspects concerning the lifecycle of neurotrophins and their receptors have been characterised over the years, including the formation, endocytosis and trafficking of signalling-competent ligand–receptor complexes. However, the molecular mechanisms directing the sorting of activated neurotrophin receptors are still elusive. Previously, our laboratory identified Bicaudal-D1 (BICD1), a dynein motor adaptor, as a key factor for lysosomal degradation of brain-derived neurotrophic factor (BDNF)-activated TrkB (also known as NTRK2) and p75^NTR^ (also known as NGFR) in motor neurons. Here, using a proteomics approach, we identified protein tyrosine phosphatase, non-receptor type 23 (PTPN23), a member of the endosomal sorting complexes required for transport (ESCRT) machinery, in the BICD1 interactome. Molecular mapping revealed that PTPN23 is not a canonical BICD1 cargo; instead, PTPN23 binds the N-terminus of BICD1, which is also essential for the recruitment of cytoplasmic dynein. In line with the BICD1-knockdown phenotype, loss of PTPN23 leads to increased accumulation of BDNF-activated p75^NTR^ and TrkB in swollen vacuole-like compartments, suggesting that neuronal PTPN23 is a novel regulator of the endocytic sorting of neurotrophin receptors.

## Abstract (precis)

**Highlighted Article:** PTPN23 associates with BICD1 via its dynein-binding region, and its loss results in accumulation of neurotrophin receptors in enlarged, ubiquitylated sorting compartments.

## INTRODUCTION

Neurotrophins (NTs) control several aspects of neuronal development, including differentiation, dendritic branching, axonal growth and axon guidance (Oppenheim, 1989; Huang and Reichardt, 2001; Ascano et al., 2012; Garcia et al., 2009). The NT family comprises nerve growth factor (NGF), brain-derived neurotrophic factor (BDNF), neurotrophin-3 (NT-3; also known as NTF3) and NT-4/5 (NTF4 and NTF5), which activate two distinct classes of receptors: catalytic tropomyosin receptor kinase (Trk) receptors, which execute trophic signalling, and the p75 nerve growth factor receptor (p75^NTR^; also known as NGFR) (Chao, 2003). NGF preferentially binds to TrkA (NTRK1), BDNF and NT-4/5 to TrkB (NTRK2) and NT-3 to TrkC (NTRK3) (Huang and Reichardt, 2001), but all NTs, in their precursor or mature form, interact with p75^NTR^ (Roux and Barker, 2002). Pro-NT binding to p75^NTR^ activates apoptosis, whilst binding of mature growth factors to this receptor promotes trophic signalling (Chao, 2003). In addition, p75^NTR^ can cooperate with Trks to form high-affinity sites for neurotrophin binding. During development, neurotrophins are made available in temporally and spatially restricted amounts, thereby determining the differentiation and survival of specific subpopulations of neurons (Ernfors, 2001). Conversely, NTs can also elicit cell death, leading to neuronal loss as part of the normal developmental process (Lanser and Fallon, 1984; Oppenheim et al., 1990; Frade et al., 1996). In addition, neurotrophins contribute to regulating neuronal plasticity, and therefore play an integral part in establishing higher functions, such as learning, memory and behaviour (Cunha et al., 2010). In adulthood, they promote neuronal homeostasis, and their withdrawal is detrimental to the health of the nervous system (Mitre et al., 2017; Yamashita and Kuruvilla, 2016; Chen et al., 2017).

Neuronal fate is controlled by the somatic integration of distally-acquired signals, such as those elicited by neurotrophins and their activated receptors (Campenot, 1977). The final cellular destination of endocytosed neurotrophin receptors (NTRs) depends on numerous factors and may ultimately lead to their recycling, transcytosis or degradation (Barford et al., 2017). Molecular motors play an important part in this process by enabling trafficking of NTRs to appropriate subcellular sites, which are specified by distinct molecular cues (Barford et al., 2017; Villarroel-Campos et al., 2018). Furthermore, ligand binding and induced post-translational modifications, including phosphorylation and ubiquitylation, also determine NTR fate and their resulting signal amplitude. As noted by Proenca et al. (2016), stimulation with NT-4, rather than BDNF, results in a more sustained signalling downstream of TrkB, as well as lower receptor ubiquitylation and degradation, although, interestingly, TrkB phosphorylation in response to both ligands is comparable.

As a result of intense research on the characterization of NTR dynamics, we currently have a broad understanding of the endocytosis, signalling, trafficking and composition of NTR carriers (Yamashita and Kuruvilla, 2016; Villarroel-Campos et al., 2018). However, there are still outstanding questions to be addressed, such as what molecular determinants are required to ensure the correct post-endocytic sorting and ultimate fate of activated NTRs. Using a medium-throughput siRNA screen, our laboratory has previously identified Bicaudal-D1 (BICD1) as a factor necessary for lysosomal downregulation of activated TrkB and p75^NTR^ in embryonic stem cell-derived motor neurons (ES-MNs) (Terenzio et al., 2014a,b). BICD1 belongs to a growing family of cytoplasmic dynein motor adaptors (Hoogenraad and Akhmanova, 2016), and facilitates various retrograde trafficking events (Matanis et al., 2002; Wanschers et al., 2007; Indran et al., 2010). However, it is an unlikely player in the long-range transport of signalling endosomes (Terenzio et al., 2014b; Reck-Peterson et al., 2018).

To further our understanding of the BICD1-dependent sorting mechanism, we characterised the BICD1 interactome using a proteomic approach, and identified protein tyrosine phosphatase, non-receptor type 23 (PTPN23; or histidine domain-containing protein tyrosine phosphatase, HD-PTP), a member of the endosomal sorting complexes required for transport (ESCRT) machinery (Tabernero and Woodman, 2018) as a BICD1 binding partner. PTPN23 directs the molecular sorting of several transmembrane receptors, such as epidermal growth factor receptor (EGFR) and platelet-derived growth factor receptor (PDGFR) in non-neuronal cells (Doyotte et al., 2008; Ma et al., 2015). In this work, we explored the relationship between BICD1 and PTPN23, and the role of PTPN23 in NTR sorting. We found that neuronal PTPN23 controls TrkB and p75^NTR^ release from early endocytic compartments, thereby establishing NTR sorting as a key function of PTPN23 in mammalian neurons.

## RESULTS

### Investigating the BICD1 interactome during endocytic sorting of NTRs

We previously identified the dynein motor adaptor BICD1 as an important player in lysosomal targeting of ligand-activated TrkB and p75^NTR^ (Terenzio et al., 2014a,b). To better understand the role of BICD1 in this process, we determined the BICD1 interactome by an immunoprecipitation approach using a BICD1-specific antibody followed by mass spectrometry. In line with our previous work (Terenzio et al., 2014a), we chose ES-MNs, which express endogenous TrkB and p75^NTR^, and a stably transfected line of mouse neuroblastoma N2A cells overexpressing FLAG-TrkB (hereafter N2A-FLAG-TrkB) (Chen et al., 2005), as our cellular models. Prior to co-immunoprecipitation, cells were either stimulated for 15 min with BDNF or kept in control medium lacking neurotrophins, as indicated in Table S1.

Our approach identified a small number of previously reported BICD1 interactors, including cytoplasmic dynein heavy chain (Matanis et al., 2002) and Fragile X mental retardation protein (FMRP) (Bianco et al., 2010), as well as several new putative binding proteins (Table S1). BICD1 interactors were ranked based on their presence in all BICD1 immunoprecipitates obtained from lysates of untreated and BDNF-stimulated ES-MNs and N2A-FLAG-TrkB cells, and characterised by gene ontology classifiers associated with intracellular transport and localisation as provided by the database for annotation, visualization and integrated discovery (DAVID, v6.8) (https://david.ncifcrf.gov/). To avoid selecting common contaminants, we consulted the contaminant repository for affinity purification (CRAPome; http://crapome.org). Among the highest ranked hits (Table 1), we selected the tumour suppressor PTPN23 (Manteghi et al., 2016; Gingras et al., 2017) for follow-up analyses based on its established role in endosomal sorting (Doyotte et al., 2008). In non-neuronal cells, PTPN23 directly regulates the function of the ESCRT machinery, which controls the biogenesis of multivesicular bodies (MVBs) and their cargo degradation in lysosomes (Ali et al., 2013; Lee et al., 2016; Woodman, 2016; Gahloth et al., 2017). Importantly, acute downregulation of PTPN23 in HeLa cells (Doyotte et al., 2008) and of BICD1 in ES-MNs (Terenzio et al., 2014a) results in a very similar phenotype, characterised by the accumulation of activated growth factor receptors in enlarged endocytic compartments, thus suggesting that PTPN23 and BICD1 regulate sorting of activated NTRs in neurons. Although the role of PTPN23 in the turnover of transmembrane receptors is well documented (Doyotte et al., 2008; Ma et al., 2015; Kharitidi et al., 2015), its function in the mammalian nervous system has not been explored to date (Gingras et al., 2009). To this end, we sought to investigate the molecular interaction between BICD1 and PTPN23, as well as the role that PTPN23 plays in the endocytic sorting of NTRs.

**Table 1.**
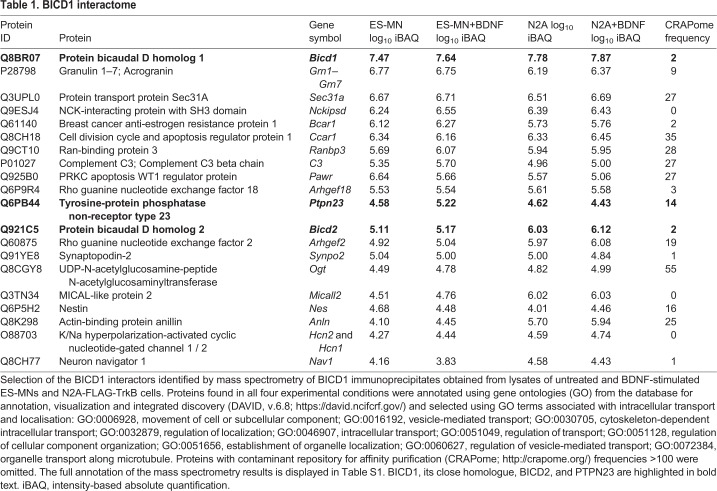
**BICD1 interactome**

### PTPN23 associates and partially colocalises with BICD1 in neuronal cells

**Fig. 1.**
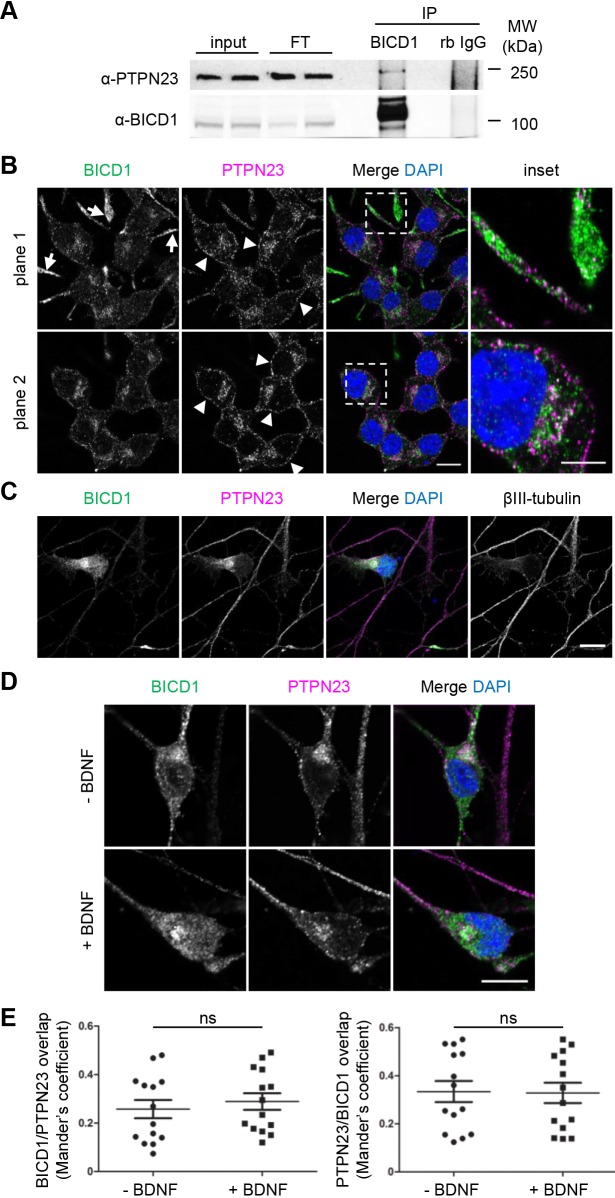
**PTPN23 co-immunoprecipitates and** **colocalises** **with BICD1 in motor neurons and in a neuronal cell line.** (A) N2A-FLAG-TrkB cell lysates were subjected to co-immunoprecipitation using Protein-G magnetic beads, pre-coated with affinity-purified anti-BICD1 polyclonal antibody or rabbit IgG as a control. Western blotting of the immunoprecipitates using anti-PTPN23 and anti-BICD1 antibodies shows specific co-immunoprecipitation of endogenous PTPN23 with BICD1 (*n*=5). (B) Confocal images of N2A-FLAG-TrkB cells, fixed and immunostained using anti-BICD1 and anti-PTPN23 antibodies. BICD1 is enriched in cell protrusions (arrows), whilst PTPN23 is enriched near the cell surface (arrowheads). BICD1 and PTPN23 are highly abundant in the perinuclear region (inset), where their immunostaining partially overlaps (*n*=6). Scale bar: 10 μm; inset: 5 μm. (C) Confocal images of ES-MNs, fixed and immunostained for BICD1, PTPN23 and the neuronal marker βIII-tubulin, showing partially overlapping and perinuclear enrichment of BICD1 and PTPN23 (*n*=3). Scale bar: 10 μm. (D) Confocal images of ES-MNs. Cells were starved and stimulated with or without BDNF for 1 h, fixed and immunostained for BICD1 and PTPN23 (*n*=2). Scale bar: 10 μm. (E) Quantification of BICD1 and PTPN23 colocalisation (from D), using Mander's coefficient. Analysis of 14 neurons per condition; *P*>0.05, Student's *t*-test (ns, not significant).

To confirm the results obtained by mass spectrometry, we performed co-immunoprecipitation experiments using N2A-FLAG-TrkB cell lysates, and assessed the colocalisation of PTPN23 and BICD1 in neuronal cells by confocal microscopy. First, the specificity of anti-PTPN23 antibody was validated in N2A-FLAG-TrkB cells by shRNA-mediated knockdown. The PTPN23 signal was significantly reduced in cells incubated with shRNAs specific for PTPN23 in comparison to scrambled shRNA-treated samples, as observed by western blotting (Fig. S1A) and immunocytochemistry (Fig. S1B). Endogenous PTPN23 was consistently isolated in BICD1 immunoprecipitates (Fig. 1A), albeit at low abundance, suggesting that the association between these proteins may be transient, or alternatively, that only specific sub-pools of these two proteins interact in neuronal cells.

Neuronal BICD1 and PTPN23 are predominantly cytoplasmic proteins displaying a punctate distribution pattern (Fig. 1B,C), suggesting that they are associated with membranous organelles (Matanis et al., 2002; Doyotte et al., 2008). In N2A-FLAG-TrkB cells, BICD1 accumulated most notably at the tips of cell protrusions (Fig. 1B, arrows), whereas PTPN23 was most abundant in the proximity of the plasma membrane (Fig. 1B, arrowheads). The highest level of colocalisation occurred in the perinuclear region of N2A-FLAG-TrkB cells (Fig. 1B) and ES-MNs (Fig. 1C,D), where the Golgi, MVBs and lysosomes also localise (Huotari and Helenius, 2011). No significant change in the colocalisation of BICD1 and PTPN23 was found upon BDNF stimulation (Fig. 1D,E), in contrast to the modulation of the levels of PTPN23 co-immunoprecipitating with BICD1 observed in the mass spectrometry experiments in ES-MNs and N2A-FLAG-TrkB cells (Table S1). A possible explanation of this unexpected result is that NTR trafficking and sorting may be differentially regulated in time and space in these cells.

The association between BICD1, the Golgi and the *trans*-Golgi network (TGN) is well documented. Most notably, BICD1, via the Golgi-associated small GTPase Rab6, facilitates the COPI-independent transport of vesicles to the endoplasmic reticulum (ER) (Matanis et al., 2002). In contrast, the relationship between PTPN23 and these organelles of the secretory pathway is less clear. To this end, neuronal cells were treated with brefeldin A (BFA), which inhibits ER-to-Golgi transport, thereby leading to progressive redistribution of the Golgi proteins back to the ER (Fujiwara et al., 1988). As expected, treatment with BFA led to the dispersal of BICD1, the Golgi and the TGN, as indicated by the distribution of their respective markers, GM130 and TGN46, respectively (Fig. S2A). However, the perinuclear staining of PTPN23 was not affected by this treatment (Fig. S2B). Although PTPN23 immunostaining partially overlapped with TGN46 in control ES-MNs, the accumulation of PTPN23 near the TGN appeared to be independent of this organelle's integrity (Fig. S2B), suggesting that, unlike BICD1, PTPN23 is not associated with the Golgi under experimental conditions.

### PTPN23 and cytoplasmic dynein bind to the N-terminal CC1 domain of BICD1

**Fig. 2.**
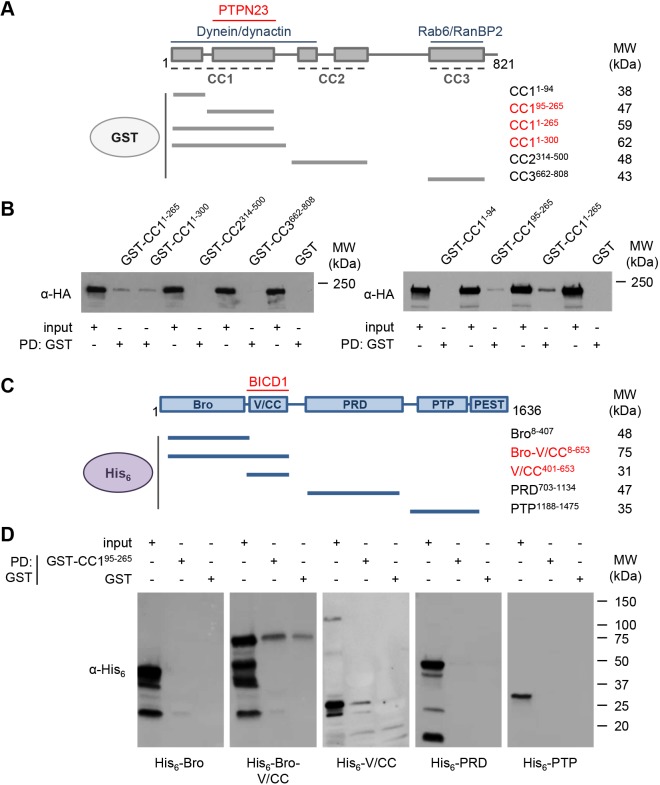
**BICD1 and PTPN23 directly interact *in vitro*.** (A) Schematic representation of BICD1 domain architecture, depicting its coiled coil domains (CC1–CC3) and highlighting the known binding regions of dynein, dynactin, Rab6 and RanBP2. Below, a schematic of GST-BICD1 fragments, with predicted molecular weights (MW; kDa). GST-BICD1 proteins in red bind HA-PTPN23 in GST pull downs. (B) GSH resin pre-loaded with GST recombinant proteins was incubated with N2A-FLAG-TrkB cell lysates, containing overexpressed HA-PTPN23 (*n*=2). Following GST pull downs, eluted proteins were assessed by immunoblotting using anti-HA antibodies. BICD1-CC1^95-265^ is the shortest fragment precipitating HA-PTPN23 (*n*=6). (C) Schematic representation of PTPN23 domain architecture, and His_6_-PTPN23 fragments used for GST pull downs, with predicted MW. His_6_-PTPN23 proteins in red bind GST-BICD1. (D) GSH resin pre-loaded with GST-CC1^95-265^ or GST was incubated with bacterial cell extracts containing His_6_-PTPN23 fragments (*n*=3). Pulled down proteins were eluted and assessed by immunoblotting using anti-His_6_ antibodies.

To identify the determinants of BICD1-PTPN23 binding at the molecular level, we used a GST pull down strategy. First, we incubated different GST-BICD1 fusion proteins (Fig. 2A) with lysates of N2A-FLAG-TrkB cells overexpressing HA-PTPN23. Based on previous results (Terawaki et al., 2015), we anticipated that PTPN23 would interact with the C-terminal CC3 domain of BICD1, an autoinhibitory region promoting the binding of cargoes, such as Rab6 and RanBP2. However, the GST-BICD1 fragments encompassing the CC2 and CC3 domains did not display any binding to HA-PTPN23. In stark contrast, we found that all GST-BICD1 recombinant proteins containing amino acids 95–265 of the CC1 domain did interact with HA-PTPN23 (Fig. 2B). This was unexpected, as the N-terminus of BICD1, which comprises the 95–265 region, when released from its autoinhibitory state upon cargo binding to the C-terminus, is known to form high-affinity interactions with cytoplasmic dynein, activating its procession along microtubules (Hoogenraad et al., 2003; Hoogenraad and Akhmanova, 2016).

To establish whether the binding between BICD1 and PTPN23 is direct, we performed GST pull downs using purified GST-CC1^95-265^ and bacterially expressed His_6_-PTPN23 fragments encompassing different functional domains (Fig. 2C). These experiments revealed a direct interaction between GST-CC1^95-265^ and the V-shaped coiled coil (V/CC) domain of PTPN23 (Fig. 2D). Whilst the interaction between GST-CC1^95-265^ and His-V/CC^401-653^ was stronger when a longer segment of PTPN23 encoding the Bro domain was expressed, we detected a significant binding of His_6_-Bro-V/CC to control GST. The association of BICD1 with the Bro and V/CC domains of PTPN23 is consistent with their established roles in cargo sorting and MVB biogenesis (Doyotte et al., 2008).

Taken together, these experiments revealed a direct interaction between BICD1 and PTPN23. Moreover, PTPN23 did not bind the CC3 domain of BICD1, and hence it may not have the ability to unlock BICD1 from its autoinhibited conformation. Importantly, the interaction of PTPN23 with the CC1 domain raises the possibility that it may compete with cytoplasmic dynein for BICD1 binding.

### GFP-BICD1^Δ95-265^ localises to the cell periphery

**Fig. 3.**
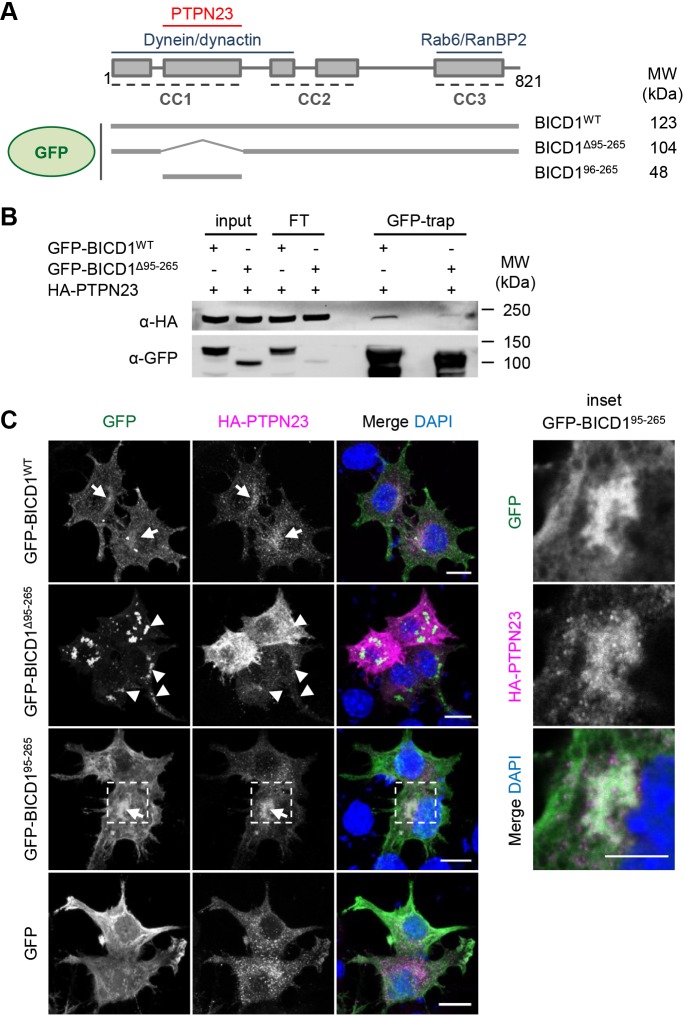
**GFP-BICD1^Δ95-265^ displays reduced association with PTPN23.** (A) Schematic of GFP-BICD1 proteins used for co-immunoprecipitation and confocal microscopy. (B) N2A-FLAG-TrkB cells were transfected overnight with plasmids encoding HA-PTPN23 and GFP-BICD1^WT^ or GFP-BICD1^Δ95-265^, and cell extracts subjected to co-immunoprecipitation using GFP-trap beads (*n*=1). Eluted proteins were assessed by immunoblotting using anti-HA and anti-GFP antibodies, revealing reduced binding between mutant GFP-BICD1^Δ95-265^ and HA-PTPN23, relative to GFP-BICD1^WT^. (C) N2A-FLAG-TrkB cells were transfected overnight with plasmids expressing HA-PTPN23 and GFP-BICD1 variants or GFP as a control, fixed and immunostained using anti-HA antibody (*n*=3). GFP-BICD1^WT^ and GFP-BICD1^95-265^ (see inset) colocalise with HA-PTPN23 (arrows), whilst the aggregation-prone GFP-BICD1^Δ95-265^ translocates to the cell periphery (arrowheads). Images show maximum intensity *Z*-stack projections, acquired at 0.5 μm spacing; insets show a representative frame selected from the respective *Z*-stack. Scale bars: 10 μm, inset: 5 μm.

To further validate our findings, we generated a BICD1 mutant lacking the PTPN23-binding domain (GFP-BICD1^Δ95-265^) (Fig. 3A). As shown in Fig. 3B, GFP-BICD1^Δ95-265^ displayed very limited binding to HA-PTPN23 in N2A-FLAG-TrkB cell lysates. This residual interaction might be due to the formation of a GFP-BICD1^Δ95-265^- BICD1^WT^ heterodimer (Terawaki et al., 2015), which has reduced PTPN23 binding compared with the BICD1^WT^ homodimer.

Next, we assessed the distribution of HA-PTPN23 and GFP-BICD1 in N2A-FLAG-TrkB cells (Fig. 3C). The punctate distribution pattern and perinuclear enrichment of HA-PTPN23 and GFP-BICD1^WT^ resembled the localisation of the endogenous proteins. In contrast, GFP-BICD1^Δ95-265^ translocated to the cell periphery, similarly to a dominant-negative BICD2-CC3 construct (Matanis et al., 2002), and was prone to aggregation (Fig. 3C). We did not, however, observe any significant colocalisation of this mutant with HA-PTPN23. In contrast, GFP-BICD1^95-265^ displayed mainly a diffused distribution, which was similar to that of GFP. However, in numerous cells we detected a perinuclear enrichment of this mutant, which overlapped with HA-PTPN23 (see Fig. 3C, inset).

Overexpressed BICD2-CC3 has been shown to have a dominant-negative effect, which leads to its accumulation, together with Rab6-positive vesicles, at the Golgi, as well as in the cell periphery (Matanis et al., 2002). Owing to the similar localisation pattern of BICD2-CC3 and GFP-BICD1^Δ95-265^ (Fig. 3C; Matanis et al., 2002), we next asked whether expression of this mutant affected the localisation of Rab6. In our experiments, GFP-BICD1^WT^ and GFP-BICD1^Δ95-265^, but not GFP-BICD1^95-265^, colocalised with Rab6-positive vesicles (Fig. S3). Furthermore, GFP-BICD1^Δ95-265^ induced a change in the localisation of Rab6-positive organelles (Fig. S3, inset) without affecting the morphology of the Golgi. These results suggest that overexpression of the GFP-BICD1^Δ95-265^ mutant, lacking a portion of the dynein-binding region, may affect the localization of endogenous Rab6 and thus potentially disrupt the retrograde trafficking controlled by this GTPase (Matanis et al., 2002; Wanschers et al., 2007).

### PTPN23 associates with TrkB-positive endocytic vesicles

Having established a PTPN23–BICD1 interaction, and in light of the role of BICD1 in NTR trafficking (Terenzio et al., 2014a,b), we hypothesised that PTPN23 might be a co-regulator of NTR dynamics. Previous findings support this hypothesis as PTPN23 was identified in the proteome of signalling endosomes isolated from ES-MNs (Debaisieux et al., 2016). Interestingly, the association of PTPN23 with signalling endosomes containing NT-NTR complexes (Deinhardt et al., 2006), increases during endosome maturation and subsequent axonal transport, suggesting that PTPN23 may play a role in trafficking and/or cargo sorting of signalling endosomes (Debaisieux et al., 2016). In addition, both BICD1 and PTPN23 were identified as potential binding partners of TrkA (Emdal et al., 2015), and a next-generation interaction survey in mammalian cells (Hein et al., 2015) found a putative association between PTPN23 and kinase D-interacting substrate of 220 kDa (Kidins220), a NTR-associated scaffolding protein (Neubrand et al., 2012).

**Fig. 4.**
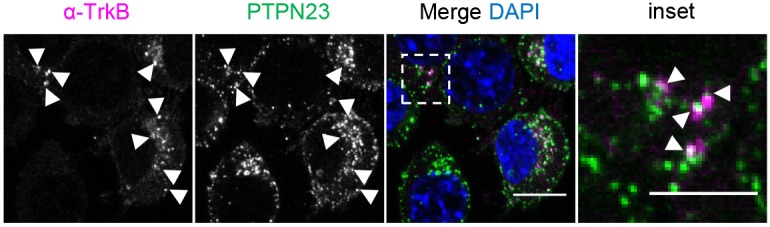
**PTPN23 associates with TrkB-containing endocytic vesicles.** Confocal images of anti-TrkB antibody accumulation assay in N2A-FLAG-TrkB cells. Cells were incubated for 30 min with antibody and chased for 30 min with 100 ng/ml BDNF. Following acid wash and fixation, cells were immunostained using anti-PTPN23 antibodies and Alexa Fluor 488-conjugated secondary antibodies. Alexa Fluor 555-conjugated secondary antibodies were used to reveal the localisation of TrkB (*n*=1). Scale bar: 10 μm; inset: 5 μm.

To test the link between PTPN23 and NTR trafficking, we performed accumulation assays of anti-NTR antibodies in N2A-FLAG-TrkB cells. Cells were incubated with antibodies directed against the extracellular portion of TrkB, followed by BDNF stimulation to promote receptor internalisation (Deinhardt et al., 2006). Importantly, these antibodies do not perturb the trafficking or signalling capacity of NTRs (data not shown). Using this approach, we detected internalised TrkB in a punctate pattern in the perinuclear region, which partially overlaps with PTPN23 (Fig. 4), suggesting that, similarly to BICD1 (Terenzio et al., 2014a), PTPN23 associates with endosomes carrying internalised TrkB in neuronal cells.

### PTPN23-deficient cells accumulate p75^NTR^ and TrkB in vacuole-like compartments

**Fig. 5.**
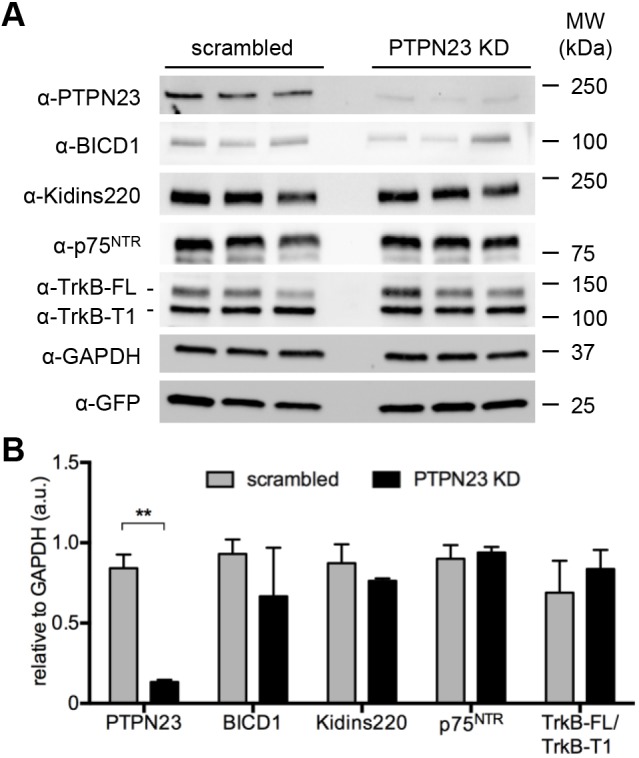
**PTPN23 is not essential to maintain steady-state levels of NTRs.** (A) N2A-FLAG-TrkB cells were transduced for 72 h with scrambled or PTPN23-targeting (PTPN23-KD) shRNAs. Cell lysates were immunoblotted for PTPN23 and NTR and related proteins of interest. GAPDH was used as a loading control, and the levels of GFP lentiviral reporter were assessed (*n*=3). (B) Densitometric analysis of protein levels from A, normalised to GAPDH (*n*=3). **P<0.01, unpaired Student's *t*-test.

To determine whether PTPN23 is required for the homeostasis of NTRs, we downregulated its expression using short hairpin RNA (shRNA). First, we chose two scrambled (scr) and three PTPN23-targeting shRNA lentiviruses, which all expressed GFP as a reporter. Transduction with two of these lentiviruses (sh1 and sh2) significantly reduced PTPN23 protein levels, which inversely correlated with the expression of the GFP reporter (Fig. S1). Because of sh2 lentivirus toxicity and off-site effects (data not shown), we chose sh1 for further work. Crucially, downregulation of PTPN23 by ∼70% did not alter the protein levels of TrkB, p75^NTR^, BICD1 and Kidins220 (Fig. 5A,B), suggesting that PTPN23 does not affect the levels of these proteins in neuronal cells.

We next ascertained whether PTPN23 downregulation alters the cellular distribution of NTRs by initially focussing our analyses on endogenous p75^NTR^, which is highly expressed in N2A-FLAG-TrkB cells. N2A-FLAG-TrkB immunostaining revealed that the abundance of p75^NTR^ on the cell surface was comparable between PTPN23-knockdown (KD) and control cells (Fig. S4A), suggesting that PTPN23 is not necessary for the steady-state sorting of p75^NTR^ to the plasma membrane or its recycling. In addition, we did not observe any overt differences in total p75^NTR^ levels (Fig. S4B), which aligned with our immunoblotting results (Fig. 5A,B).

**Fig. 6.**
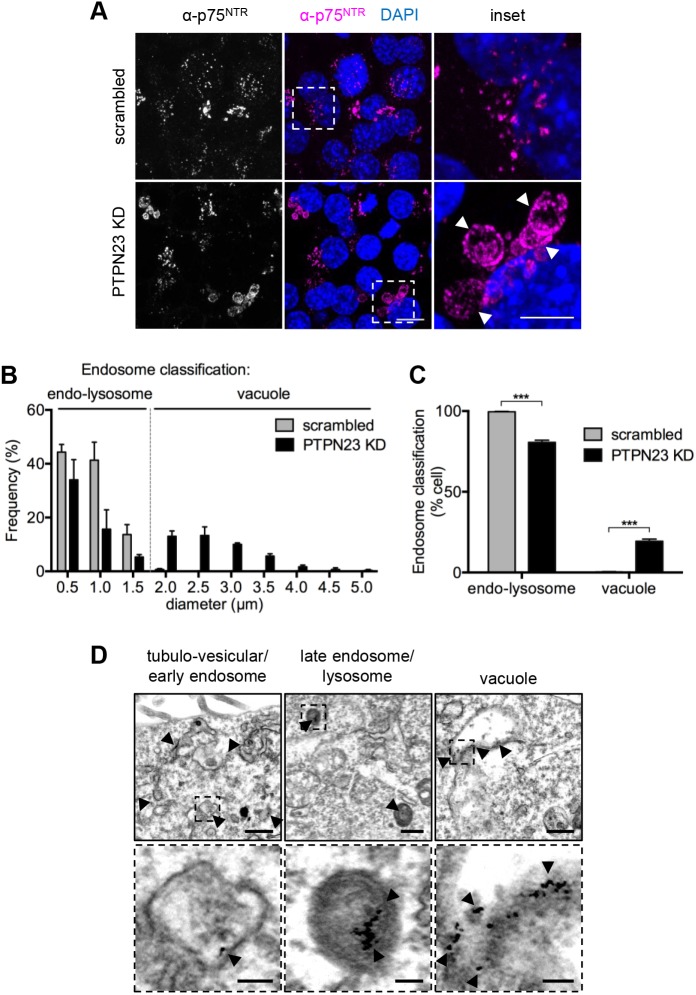
**Silencing of PTPN23 leads to p75^NTR^ accumulation in vacuole-like compartments.** (A) Confocal images of α-p75^NTR^ accumulation assay in scrambled and PTPN23 shRNA treated N2A-FLAG-TrkB cells (*n*=3). Cells were incubated for 30 min with anti-p75^NTR^ antibody and chased for 30 min with 100 ng/ml BDNF. Following acid wash and fixation, receptor localisation was revealed using Alexa Fluor 555-conjugated secondary antibody. Arrowheads indicate enlarged endocytic compartments. Images show maximum intensity *Z*-stack projections, acquired at 0.5 μm spacing. Scale bar: 10 μm; inset: 5 μm. (B) Diameters of 100 endosomes (per *n*/condition) were measured and grouped using 0.5 μm binning, in three independent experiments. Endosomes were classified as endo-lysosomes (<1.5 μm) or vacuoles (>1.5 μm). ****P<0.0001, χ*^2^* (two-tailed chi-square) test for trend (*n*=3). (C) Quantification of cells containing endo-lysosomes or vacuole-like compartments (*n*=3); cells lacking α-p75^NTR^ accumulation (∼50% of cells) were counted but excluded from the analysis. ***P<0.001, unpaired Student's *t*-test. (D) Transmission electron microscopy images showing three representative classes of organelles containing accumulated α-p75^NTR^-gold (arrowheads). Early and late endocytic compartments were indistinguishable between scrambled and PTPN23-KD cells, and representative images taken from PTPN23-KD and scrambled cells, respectively; vacuoles were observed only in PTPN23-KD cells. Scale bars: 500 nm; insets: 100 nm.

To determine whether PTPN23 downregulation affects the endocytic sorting of p75^NTR^, we performed an anti-p75^NTR^ antibody (α-p75^NTR^) accumulation assay. In control cells, we detected a punctate distribution of α-p75^NTR^ predominantly in the perinuclear region (Fig. 6A), a pattern which closely resembled that of TrkB (Fig. 4). However, in PTPN23-KD cells, in addition to α-p75^NTR^ puncta, we observed enlarged organelles with α-p75^NTR^ labelling in their limiting membrane (Fig. 6A). Similar observations were made for FLAG-TrkB, following an accumulation assay using an anti-FLAG antibody (Fig. S5A). This vacuolar phenotype aligns well with previous studies reporting the role of PTPN23 in receptor sorting in non-neuronal cells (Doyotte et al., 2008; Ma et al., 2015), and with the NTR accumulation phenotype displayed by BICD1-depleted ES-MNs (Terenzio et al., 2014a).

To assess the effect of PTPN23 downregulation on the morphology of NTR-containing endosomes, we measured the diameters of endocytic organelles containing α-p75^NTR^ in control and sh1-treated cells. The size distribution of α-p75^NTR^-labelled endo-lysosomes (<1.5 µm in diameter) and vacuoles (>1.5 µm in diameter) was significantly different between PTPN23-KD and control cells (****P<0.0001; χ*^2^* test for trend; Fig. 6B), with some of the vacuoles reaching 5 μm in diameter in cells depleted of PTPN23. Strikingly, these large compartments were detected only in 20% of PTPN23-KD cells (Fig. 6C), thus suggesting that residual PTPN23 (Fig. S1) or an alternative mechanism contributes to p75^NTR^ sorting in these conditions.

Different populations of organelles containing endocytosed NTRs were further cross-examined by transmission electron microscopy (Fig. 6D, Fig. S5B). Gold nanoparticles conjugated to the anti-p75^NTR^ antibody (α-p75^NTR^-gold) were detected predominantly in membranous and tubular compartments (Fig. 6D, Fig. S5B), reminiscent of early endosomes. In addition, these antibodies revealed that p75^NTR^ accumulated in late endosomes and lysosomes (Fig. 6D). Whilst these organelles were also detected in control and PTPN23-KD cells, α-p75^NTR^-gold-labelled vacuoles with a diameter larger than 1.5 µm were observed exclusively in cells depleted of PTPN23 (Fig. 6D, Fig. S5B). These swollen compartments were devoid of internal vesicles akin to those normally seen in MVBs, and accumulated α-p75^NTR^-gold near their surrounding membrane, suggesting that loss of PTPN23 resulted in defective sorting of NTRs and potentially other cargoes associated with these organelles.

Taken together, our findings imply that PTPN23 is required for the endocytic trafficking of NTRs, and its depletion in neuronal cells caused endosomal swelling. Crucially, the accumulation of α-p75^NTR^ in vacuoles was rescued by PTPN23 overexpression (Fig. S6), confirming that the observed phenotype is a direct consequence of PTPN23 loss.

### p75^NTR^ in vacuoles is heavily ubiquitylated

**Fig. 7.**
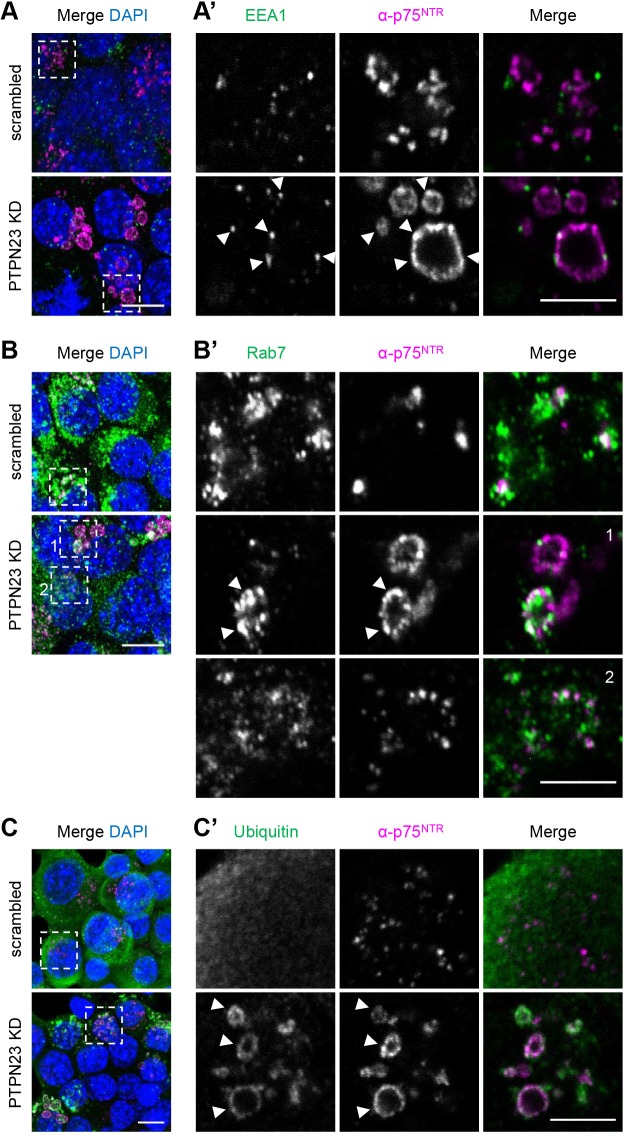
**Vacuolar compartments, containing α-p75^NTR^, are sorting endosomes enriched in ubiquitylated proteins.** Confocal images of α-p75^NTR^ accumulation in scrambled and PTPN23 shRNA-treated N2A-FLAG-TrkB cells. Following acid wash and fixation, cells were immunostained using anti-EEA1 (A), anti-Rab7 (B) and anti-ubiquitin (C) antibodies (*n*=3). Arrowheads indicate co-localization of these proteins within enlarged endocytic compartments. (A,B,C) Merged images showing maximum intensity Z-stack projections, acquired at 0.5 μm spacing. (A′,B′,C′) Insets show representative frames selected from corresponding Z-stacks in A,B,C, respectively. Scale bars: 10 μm; insets: 5 μm.

To better understand the identity of the enlarged endosomal compartments containing α-p75^NTR^ (Fig. 6, Fig. S5B), we performed an immunofluorescence staining for early and late endosomal markers. In control cells, no significant colocalisation between α-p75^NTR^ and the early endosomal marker EEA1 was detected (Fig. 7A). In contrast, EEA1 was detected in α-p75^NTR^-containing organelles in PTPN23-KD cells (Fig. 7A). Interestingly, EEA1 displayed a punctate staining pattern on the limiting membrane of the enlarged α-p75^NTR^-positive endosomal compartments.

In contrast, α-p75^NTR^ puncta colocalised with Rab7 in both PTPN23-KD and control cells (Fig. 7B), confirming that internalised p75^NTR^ progresses to late endocytic organelles en route to lysosomes. Interestingly, only a small proportion of the vacuoles found in PTPN23-KD cells accumulated Rab7 (Fig. 7B, inset 1).

Taken together, our findings suggest that in cells depleted of PTPN23, α-p75^NTR^ accumulates in hybrid organelles containing early endosomal markers, which is suggestive of an intermediate sorting compartment (Poteryaev et al., 2010; Barford et al., 2017). The massive endosomal enlargement driven by PTPN23 downregulation suggests that PTPN23 is necessary for endosome maturation, with its depletion causing the disruption of endocytic flow, consequent endocytic swelling and receptor retention in dysfunctional sorting compartments (Huotari and Helenius, 2011).

Lastly, we assessed whether the enlarged α-p75^NTR^-containing compartments observed after PTPN23 depletion accumulated ubiquitin. Although p75^NTR^ ubiquitylation has not been extensively explored to date (Sánchez-Sánchez and Arévalo, 2017), stimulation of the hippocampal HT-22 cell line with NGF results in phosphorylation-dependent p75^NTR^ ubiquitylation by the ubiquitin ligase TRAF6 (Geetha et al., 2012). Whilst ubiquitin often marks its cargo for degradation, deubiquitylation is a prerequisite for cargo degradation to proceed. Crucially, PTPN23 enables this ubiquitin recycling step by recruiting the deubiquitylase USP8 onto EGFR (Ali et al., 2013). Here, we observed that all enlarged endocytic compartments found in cells depleted of PTPN23 accumulate ubiquitin, albeit at different levels (Fig. 7C). Whilst the ubiquitylation levels of p75^NTR^ were not assessed directly, increased TrkB ubiquitylation was previously detected in motor neurons depleted of BICD1 (Terenzio et al., 2014a), and elevated EGFR-containing vacuole ubiquitylation was detected in HeLa cells upon PTPN23 silencing (Doyotte et al., 2008).

## DISCUSSION

A correct balance between receptor degradation and recycling ensures the precise responses of downstream signalling effectors to extracellular cues. This process is tightly regulated at several levels, from initial receptor endocytosis upon ligand binding, through to endocytic transport and sorting of activated receptors to appropriate subcellular destinations (Huotari and Helenius, 2011). Accurate spatiotemporal regulation of these intracellular trafficking steps and the associated signalling responses elicited by neurotrophins is particularly important for neurons, and ensures the correct development of the nervous system and its homeostasis in adulthood (Bronfman et al., 2007). It is therefore not surprising that deregulation of NTR signalling is associated with neurological disorders (Bilsland et al., 2010; Gupta et al., 2013; Simmons, 2017). Whilst several aspects of NTR signalling and intracellular transport have been characterised (Villarroel-Campos et al., 2018), the machinery regulating the endocytic sorting of ligand-activated NTRs is not yet completely understood.

With the aim of identifying new regulators of NTR sorting, the present work explored the interactome of BICD1, a cytoplasmic dynein adaptor previously found to be essential for the downregulation of ligand-activated TrkB and its signalling output (Terenzio et al., 2014a). Several novel putative BICD1 binding partners were identified (Table 1 and Table S1), and PTPN23 was selected for further studies. Previous work has demonstrated the role of this ESCRT-interacting protein in the sorting of several transmembrane proteins, including EGFR (Doyotte et al., 2008), PDGFR (Ma et al., 2015), α5β1 integrin (Kharitidi et al., 2015) and major histocompatibility complex class-I (MHC-I) (Parkinson et al., 2015). However, the function(s) of PTPN23 has not been explored in neurons to date, although its high expression in the brain and spinal cord predominantly at early developmental stages (Gingras et al., 2009) suggest that it plays an important role in the development of the nervous system, when neurotrophin signalling is particularly critical. In support of this, knockout of PTPN23 in mice leads to early embryonic lethality (Gingras et al., 2009). *PTPN23* mutations were recently linked to developmental epileptic encephalopathy with hypomyelination, brain atrophy and developmental delay (Alazami et al., 2015; Sowada et al., 2017; Smigiel et al., 2018), indicating that functional PTPN23 is also essential for the development of the human nervous system. Studies on Myopic, the *Drosophila* orthologue of PTPN23, suggest that its function in the nervous system may extend beyond receptor sorting, as Myopic plays a role at the neuromuscular junction by downregulating the release of insulin-like peptide 2 (Dilp-2) from dense core vesicles (Bulgari et al., 2018), as well as in synaptic pruning (Loncle et al., 2015).

Here, we demonstrated the binding of PTPN23 to BICD1 and detected their partial colocalisation in neuronal cells (Fig. 1). However, the low level of overlap suggests that these proteins may interact only transiently. In contrast to BICD1, PTPN23 does not associate with the Golgi (Fig. S2), although a pilot study identified PTPN23 as a potential player in the endosome-to-Golgi retrieval pathway (Breusegem and Seaman, 2014).

Further *in vitro* studies revealed an unusual binding mode between PTPN23 and BICD1 via a small N-terminal portion of BICD1 (amino acids 95–265). This result was unexpected (Fig. 2A,B), since the N-terminus of BICD1 is essential for recruitment of cytoplasmic dynein, whereas the C-terminus of BICD1 and related BICD proteins function as a ‘cargo-binding domain’ (Carter et al., 2016; Hoogenraad and Akhmanova, 2016). This finding highlights that PTPN23 is not a canonical BICD1 cargo, and might not have the ability to release BICD1 from its autoinhibited conformation. In addition, PTPN23 and cytoplasmic dynein could compete for BICD1 binding at the N-terminus; and since BICD1 does not appear to be the main dynein adaptor responsible for trafficking of signalling endosomes (Reck-Peterson et al., 2018), is it plausible that BICD1 function extends beyond dynein-mediated trafficking. Furthermore, binding of PTPN23 to BICD1 might be highly context specific and restricted to specific subcellular domains, subpopulations of cells or developmental stages. Alternatively, interaction between BICD1 and PTPN23 may promote the association between the trafficking machinery and specific endosomal components, facilitating their delivery to a particular destination, such as lysosomes for degradation. A future priority therefore would be to determine the relationship between PTPN23, BICD1 and dynein.

BICD1 directly binds the V/CC domain of PTPN23 (Fig. 2), which, together with the Bro domain, plays a central role in EGFR downregulation and formation of intraluminal vesicles in MVBs (Doyotte et al., 2008; Tabernero and Woodman, 2018). These domains are structurally related to those found in Alix, which is required for ESCRT function (Tabernero and Woodman, 2018). Therefore, the association of BICD1 with the V/CC domain of PTPN23 further supports a direct role of BICD1 in NTR sorting (Terenzio et al., 2014a).

To test this hypothesis, we assessed the effect of PTPN23 downregulation on the endocytic trafficking of NTRs. Antibody feeding assays revealed that PTPN23 co-distributes with endocytic vesicles containing BDNF-activated TrkB and p75^NTR^ (Fig. 4) and is required for their maturation along the endocytic pathway (Fig. 6, Fig. S5A). Upon PTPN23 knockdown, TrkB and p75^NTR^ accumulate in abnormal endocytic compartments, which have hallmarks of early endosomes or sorting organelles (Fig. 6 and Fig. 7A,B, Fig. S5B) and are heavily ubiquitylated (Fig. 7C), in agreement with the requirement of PTPN23 for mediating the recruitment of USP8 to EGFR carriers prior to receptor degradation (Doyotte et al., 2008; Ali et al., 2013). The possibility that PTPN23 recruits deubiquitylating enzymes, such as USP8, to p75^NTR^ and TrkB, is intriguing and might be explored in future studies, as it is likely to contribute to our understanding of how ubiquitylation regulates the fate of internalised NTRs (Sánchez-Sánchez and Arévalo, 2017). In support of this view, NGF-activated TrkA and USP8 were shown to interact on early endosomes in PC12 cells (Ceriani et al., 2015), and activity-dependent ubiquitylation of p75^NTR^ was also previously reported (Geetha et al., 2012). Furthermore, several USP family members, including USP3 and USP5, associate with axonal signalling endosomes (Debaisieux et al., 2016).

Whilst enlarged endocytic organelles were observed in non-neuronal cells (Doyotte et al., 2008; Ma et al., 2015), the sorting phenotype after PTPN23 silencing seems to be receptor specific. Whereas EGFR displayed an increased association with the early endocytic marker EEA1 as well as increased recycling to the plasma membrane in cells depleted of PTPN23 (Doyotte et al., 2008), no significant co-distribution of PDGFR with early and recycling endosomes has been reported under the same experimental conditions (Ma et al., 2015). In addition, silencing of PTPN23 resulted in reduced PDGFR ubiquitylation in response to ligand stimulation, even though loss of PTPN23 led to defects in receptor degradation in all cases (Doyotte et al., 2008; Ma et al., 2015).

Linking PTPN23 and BICD1 to endocytic sorting of NTRs is mechanistically challenging in light of the functional heterogeneity of signalling endosomes (Villarroel-Campos et al., 2018) and the emerging differences in how this process is regulated in post-mitotic neurons versus proliferating cells. Although further work is necessary to fully characterise the PTPN23-KD phenotype described here, the role of PTPN23 in intracellular cargo sorting is somewhat easier to interpret than how BICD1 performs this function, primarily due to the extensive literature focussed on the characterisation of PTPN23 in EGFR dynamics (Woodman, 2016; Gahloth et al., 2016, 2017; Tabernero and Woodman, 2018). Interestingly, PTPN23 binds endophilin A1 (Ichioka et al., 2007), which is involved in the endocytic sorting of TrkB (Burk et al., 2017). Endophilin knockdown leads to accumulation of TrkB in EEA1- and Rab7-positive endosomes (Burk et al., 2017), which aligns well with our results (Fig. 7). The phenotype induced by knockdown of BICD1 or PTPN23 is characterised by increased NTR accumulation in enlarged endosomes, perturbed endosome maturation, increase in ubiquitylation and receptor recycling to the plasma membrane, which overall, is in agreement with perturbed function of the ESCRT machinery (Frankel and Audhya, 2018). It is therefore tempting to speculate that BICD1 may contribute to ESCRT function at sorting endosomes, and perhaps also to MVB biogenesis, as decreased abundance of gold-conjugated HcT within MVBs was observed in motor neurons depleted of BICD1 (Terenzio et al., 2014a). In future studies, it would be interesting to determine whether loss of BICD1 leads to similar perturbations in the sorting of receptors modulated by PTPN23, such as EGFR. A similar relationship has been previously demonstrated between another dynein adaptor, Rab-interacting lysosomal protein (RILP) and the ESCRT-II components VPS22 and VPS36, leading to the hypothesis that RILP, similarly to BICD1, participates in MVB biogenesis (Progida et al., 2006; Wang and Hong, 2006), in addition to late endosome to lysosome trafficking (Reck-Peterson et al., 2018). Interestingly, both overexpression (Wang and Hong, 2006) and depletion (Progida et al., 2007) of RILP caused prolonged EGFR retention in enlarged early endosomes and perturbed MVB biogenesis in HeLa cells. Hence, it is plausible that BICD1 and PTPN23 play a similar role in MVB biogenesis in developing neurons (Terenzio et al., 2014a) and modulate both local and long-term signalling in physiological and pathological conditions (e.g. in response to injury) in the adult nervous system.

## MATERIALS AND METHODS

### Cells and reagents

Mouse embryonic stem (ES) cells were derived from hybrid blastocysts generated at the Crick Institute Biological Resource Unit, by mating C57BL6/6J and 129 (S6)SvEv mice, as previously described (Bryja et al., 2006). Mouse ES cells were maintained and differentiated into motor neurons (ES-MNs) as previously described (Terenzio et al., 2014a). Mouse neuroblastoma Neuro-2a (N2A) cell line stably expressing TrkB with N-terminal FLAG-tag (N2A-FLAG-TrkB) was described in Terenzio et al., 2014a and tested in the Cancer Research UK London Research Institute Cell Facility for contamination. All chemicals were purchased from Sigma, unless stated otherwise. Reagents for mammalian cell culture were obtained from Gibco. Reagents for polymerase chain reaction and cloning were sourced from New England Biolabs (NEB). Transfection reagents and primers were purchased from Invitrogen.

### Antibodies

The following primary antibodies were used in the study (AA, accumulation assay; ICC, immunocytochemistry; WB, western blotting): chicken anti-βIII-tubulin (#ab41489; Abcam; 1:500 ICC); rabbit anti-BICD1 (#HPA041309; Atlas Antibodies; 1:500 ICC, 1:1000 WB); mouse anti-EEA1 (#E41120; Transduction Labs; 1:50 ICC); mouse anti-FLAG (M1; #F3040; Sigma; 1:500 AA); mouse anti-GAPDH (#mab374; EMD Millipore; 1:5000 WB); mouse anti-GFP (4E12/8; CRUK; 1:2000 WB); chicken anti-GFP (#1010; Aves Labs; 1:1000 ICC); mouse anti-GM130 (#61082; BD Biosciences; 1:1000 ICC); rat anti-HA (F310, #11867423001; Roche; 1:500 ICC, 1:1000 WB); mouse anti-His_6_ (#707996-3MM; Novagen; 1:1000 WB); rabbit anti-Kidins220 (KNA; CRUK; 1:1000 WB); rabbit anti-p75^NTR^ (CRD5410; 1:1000 AA, 1:1000 ICC; Deinhardt et al., 2007); rabbit anti-p75^NTR^ (poly18397, #839701; BioLegend; 1:1000 WB); mouse anti-PTPN23 (F-4, #sc-398711; Santa Cruz; 1:400 ICC, 1:500 WB); rabbit anti-Rab6 (#9625; Cell Signaling; 1:400 ICC); mouse anti-Rab7 (#sc-376362; Santa Cruz; 1:1000 ICC); rabbit anti-TGN46 (#ab16059; Abcam; 1:100 ICC); rabbit anti-TrkB (#9872; Merck Millipore; 1:1000 AA); rabbit anti-TrkB (#07-225; Merck Millipore; 1:1000 WB); mouse anti-ubiquitin (FK2, #BLM-PW8810; Enzo Life Sciences; 1:100 ICC).

### Cell culture and transfection

N2A-FLAG-TrkB cells were cultured in Dulbecco's Modified Eagle's Medium (DMEM) supplemented with 10% fetal bovine serum (FBS) and 1% GlutaMAX. Cells were maintained in humidified incubator at 37°C, supplemented with 5% CO_2_, and passaged using 0.25% trypsin upon reaching 80% confluency. Where indicated, cells were transfected overnight using Lipofectamine^®^ 3000, according to manufacturer's instructions. For PTPN23 knockdown, N2A-FLAG-TrkB cells were transfected with shRNA plasmids for 72 h.

### Lentivirus production and cell transduction

ShRNAs directed against PTPN23 (MSH025913-LVRU6GP) and non-targeting shRNA (CSHCTR001-LVRU6GP) were obtained from the OmicsLink™ shRNA clone collection (GeneCopoeia). ShRNA in psiLVRU6GP vector was expressed under U6 promoter, and reporter eGFP under SV40. Lentiviral particles were prepared as previously described (Gibbs et al., 2018). N2A-FLAG-TrkB cells were transduced immediately after seeding, and assayed after 72 h.

### Immunocytochemistry and confocal microscopy

N2A-FLAG-TrkB cells or ES-MNs were seeded onto poly-L-lysine- or polyornithine and laminin-coated coverslips, respectively, and maintained in culture for 2–3 days. Following PBS wash and fixation with 4% paraformaldehyde (PFA) for 15 min at room temperature, cells were permeabilised with 0.1% Triton X-100 in PBS for 10 min, and blocked with 10% goat serum and 0.5% BSA in PBS for 1 h. Cells were then incubated with primary antibodies diluted in reduced blocking solution (5% goat serum, 0.5% BSA, PBS) overnight at 4°C, washed and incubated with Alexa Fluor 488-, 555 or 647-conjugated secondary antibodies (1:1000; Life Technologies) and DAPI (4′,6-diamidino-2-phenylindole) nuclear stain for 2 h at room temperature. Coverslips were mounted using mounting medium (#S3023; Dako). Images were taken on LSM510 inverted laser scanning confocal microscope (Zeiss), using 63×/1.40 oil objective.

For the analysis of BICD1 and PTPN23 colocalisation in ES-MNs, a theoretical point spread function was generated using Diffraction PSF 3D plugin in ImageJ, and images were later deconvoluted using Parallel Spectral Deconvolution 2D plugin. Co-localisation analysis was carried out with Coloc2 plugin, which provides Mander's M1 and M2 coefficients, used later for statistical analyses.

### Anti-receptor antibody accumulation assay

N2A-FLAG-TrkB cells were washed twice with DMEM and then serum-starved for 3 h. Cells were incubated with rabbit anti-TrkB (1:1000; #9872; Millipore), rabbit anti-p75^NTR^ (1:1000; CRD5410, CRUK) or mouse anti-FLAG (1:500, M1; #F3040; Sigma) antibodies for 30 min at 37°C, followed by 30 min with BDNF (100 ng/ml) to promote the internalisation of antibody-receptor complexes. As negative controls, cells were incubated with species-matched IgGs. Next, cells were pre-chilled on ice for 5 min, washed with ice-cold 0.2 M acetic acid, 0.5 M NaCl, pH 2.4 for 1 min (for anti-TrkB and anti-p75^NTR^) or magnesium and calcium-free PBS supplemented with 1 mM EDTA (for anti-FLAG), rinsed with PBS and fixed for 15 min with 4% PFA. Following permeabilization and blocking, internalised antibodies were revealed by incubation with Alexa Fluor 555-conjugated anti-rabbit (1:1000) or anti-mouse (1:500) secondary antibodies.

For endosome diameter distribution and classification analyses, five fields per sample per condition (PTPN23-KD or scrambled) were imaged by confocal microscopy using a 63× objective. Diameters of 100 endosomes were measured in Fiji (with selection criteria of 50 ‘large’ and 50 ‘small puncta-like’) in three independent experiments. Endosomes were classified, using 0.5 μm binning, as endo-lysosomes (diameter <1.5 μm) and vacuoles (1.51–5 μm). To establish the proportion of cells with either phenotype, 5–7 fields were imaged per condition in three independent experiments at 1× magnification using 63× objective, and cells containing either endo-lysosomes or vacuoles, as well as total number of cells per field (average of 80 cells per field) were counted.

### Transmission electron microscopy

Anti-p75^NTR^ antibody (CRD5410, CRUK) was conjugated to 5 nm colloidal gold nanoparticles (British Biocell; 0.9 mg/ml), as previously described (Terenzio et al., 2014a). Serum-starved N2A-FLAG-TrkB cells were incubated with anti-p75^NTR^-gold (1:500), as described above. Following washes with acid, PBS and DMEM, cells were fixed with 4% PFA in Sorensen's phosphate buffer for 15 min and post-fixed with 2.5% glutaraldehyde and 4% PFA in Sorensen's phosphate buffer for 20 min at room temperature and processed for electron microscopy as previously described (Terenzio et al., 2014a). Grids were scanned for the presence of gold, and equal number of images was obtained for all conditions. All gold-containing internal structures were imaged and classified [scrambled: 77 organelles in 37 images (52% tubulo-vesicular, 48% late endosomes/lysosomes); PTPN23 KD: 76 organelles in 39 images (39% tubulo-vesicular, 47% late endosomes/lysosomes, 13% vacuoles)].

### Co-immunoprecipitation and GFP-trap

N2A-FLAG-TrkB cells were grown to 80% confluency in 10 cm dishes and serum-starved for 3 h, as described above. Next, cells were washed with ice-cold PBS on ice, and proteins (2–4 mg) were extracted in 0.4% NP-40 lysis buffer (50 mM Tris-HCl, pH 7.5, 150 mM NaCl, 1 mM EDTA, 0.4% NP-40, 5% glycerol), supplemented with Halt™ protease and phosphatase inhibitor cocktail (1:100; Thermo Scientific). Next, 20 μl of pre-washed (0.02% Tween-20 in PBS; PBST) magnetic Dynabeads^®^ Protein G (Novex) were incubated for 30–60 min at room temperature with 2 μg anti-BICD1 antibodies or rabbit IgG, resuspended in 200 μl PBST. Following removal of unbound antibodies, beads were incubated with freshly extracted cell lysate for 2 h at 4°C.

Magnetic green (GFP)-Trap^®^ M beads (Chromotek) were used to precipitate GFP-tagged recombinant proteins, according to the manufacturer's instructions. Briefly, 25 μl of beads were equilibrated in GFP-bead wash buffer (10 mM Tris-HCl, pH 7.5, 150 mM NaCl, 0.5 mM EDTA) and 0.2% NP-40 lysis buffer, followed by incubation with freshly prepared N2A-FLAT-TrkB cell lysate containing overexpressed GFP-tagged recombinant proteins, extracted in 0.2% NP-40 lysis buffer, for 2 h at 4°C.

Dynabeads^®^/GFP-Trap^®^ M beads were washed 4× with 200 μl 0.4% NP-40 lysis buffer, transferred to fresh tubes and washed again. Proteins were eluted by boiling for 4 min at 95°C in 20 μl 1× Laemmli sample buffer (LSB). Entire eluted fraction was assessed by SDS-PAGE and western blotting. For input and flow through (FT), 1/50 of pre or post co-IP lysate was loaded, respectively.

### Co-immunoprecipitation and mass spectrometry

ES-MNs and N2A-FLAG-TrkB cells were serum-starved for 3 h in Neurobasal and DMEM, respectively, and stimulated with/without 100 ng/ml BDNF for 15 min. Cell extracts were prepared in 1% NP40 IP buffer (10 mM Tris-HCl, pH 7.5, 150 mM NaCl, 1% NP40, 1 mM EDTA), supplemented with Halt™ protease and phosphatase inhibitor cocktail (1:100; Thermo Fisher) and incubated with anti-BICD1 antibodies pre-bound to magnetic Protein-G Dynabeads^®^ as described above. Eluted fractions were subjected to SDS-PAGE, the resultant polyacrylamide gel was fixed and stained with Colloidal Blue (Thermo Fisher). Lanes were then cut into small sections and subjected to overnight in-gel digestion. Peptide mixtures were analysed using an LTQ-Orbitrap-XL mass spectrometer. Raw data was processed using MaxQuant 1.6.03 with *M. musculus* as the reference proteome and intensity based absolute quantification (iBAQ). Further annotation such as Gene Ontology and CRAPome frequencies (Mellacheruvu et al., 2013) was added to the protein groups table using Perseus version 1.4.02.

### Cloning

BICD1 and PTPN23 fragments used in GST pull downs were generated by FastCloning (Li et al., 2011). Briefly, target DNA (‘insert’) was amplified by PCR using a primer pair containing 9–15 bp overhang (in bold in Table S2), complementary to desired site on the acceptor vector. The acceptor vector was amplified using a pair of primers containing no overhangs and inclusive of the region complementary to overhang sequences on insert primers. All fragments were amplified using Phusion^®^ High Fidelity DNA polymerase (denaturation: 10 s at 98°C; annealing: 30 s at 55–68°C; extension: 20 s/kb at 72°C; 25 cycles), according to the manufacturer's instructions (NEB). Next, PCR products were diluted in water (1:3), treated with *Dpn*I for 2 h at 37°C, mixed (3:1, insert:vector) and transformed into XL-10 Gold ultracompetent *E. coli* (Stratagene).

For bacterial protein expression, BICD1 fragments were subcloned into pGEX-4T-1 vector using human BICD1 cDNA (835 aa, Q96G01-4, NM_001003398) in a pEGFPN1 vector as a source material. PTPN23 fragments were cloned into pET28a+ vector using HA-PTPN23-pcDNA3.1+ plasmid containing human PTPN23 cDNA (1636 aa, Q9H3S7-1, NM_015466.3) as described in Doyotte et al. (2008) as a template. For mammalian protein expression, GFP-BICD1 constructs were generated by FastCloning. The deletion construct GFP-BICD1^Δ95-265^ was prepared using a pair of overlapping primers (Table S2), containing *Eco*RI restriction site (underlined).

### Recombinant protein expression and purification

Recombinant GST-BICD1 and His_6_-PTPN23 fragments were expressed in SoluBL21™ *E. coli* (Amsbio), induced with 1 mM isopropyl β-D-1-thiogalactopyranoside (IPTG) at 0.4 OD_600_ overnight at 21°C in M9 minimal media (0.6% w/v Na_2_HPO_4_, 0.3% w/v KH_2_PO_4_, 0.05% w/v NaCl, 0.1% w/v NH_4_Cl, 100 mM CaCl_2_, 1 M MgSO_4_, 0.3% glycerol). Molecular weights of recombinant proteins were determined using ProtParam online prediction tool (https://web.expasy.org/protparam).

For GST-BICD1 fragments purification, bacteria were harvested by centrifugation at 3000 RPM for 10 min. Unless stated otherwise, all following steps were performed at 4°C. Pellets were washed twice with PBST and sonicated (3×20 s pulse, with 1 min cooling interval; Soniprep 150 Ultrasonic disintegrator, MSE) in GST lysis buffer (0.05% Tween20, 2 mM EDTA, 0.1% β-mercaptoethanol, 1 mM benzamidine, 0.5 mM PMSF in PBS). Insoluble material was pelleted by centrifugation for 20 min at 14,900 rpm. GST fusion proteins were purified using glutathione (GSH)-agarose affinity resin, rotating end-over-end for 2 h at 4°C. Resin was washed 3×10 beads volume with 0.05% PBST, and once with 0.05% PBST containing 0.5 M NaCl.

His_6_-PTPN23 fragments were extracted by sonication in lysis buffer (50 mM NaH_2_PO_4_, pH 8.0, 300 mM NaCl, 10 mM imidazole, 0.1% β-mercaptoethanol, 1 mM benzamidine, 0.5 mM PMSF), as described above.

### GST-BICD1 pull downs

GST-BICD1 fusion proteins pre-bound to GSH-resin were incubated for 2 h at 4°C with fresh N2A-FLAG-TrkB cell lysates containing overexpressed HA-PTPN23 in 0.4% NP-40 lysis buffer, or with bacterial extracts containing equal amounts of His_6_-PTPN23 fragments in lysis buffer. Next, resin was gently washed 4–6 times with appropriate buffer, pelleted, transferred to a fresh tube and washed again. Proteins were eluted by boiling for 4 min at 95°C in 1× LSB, and whole fraction was assessed by SDS-PAGE and western blotting using anti-HA or anti-His_6_ antibodies. For inputs, 1/50 of N2A-FLAG-TrkB cell lysate or 1/25–1/50 of bacterial lysate containing His_6_-PTPN23 fragments was loaded.

### Western blotting

Proteins were separated by 4–12% NuPAGE Bis-Tris (Novex) or 4–15% Mini-PROTEAN^®^ TGX Stain-Free™ (Bio-Rad) gels and transferred onto methanol-activated polyvinylidene fluoride (PVDF, Bio-Rad) membranes, according to the manufacturer's instructions. Membranes were blocked in 5% fat-free dry milk dissolved in PBST for 1 h at room temperature, and incubated with primary antibodies, diluted in blocking solution, for 1 h at room temperature or overnight at 4°C. Following washes with PBST, membranes were incubated with appropriate horseradish peroxidase-conjugated secondary antibodies (1:1000; Dako) for 1 h at room temperature. Next, blots were incubated with enhanced chemiluminescent substrate (Millipore), and developed using ChemiDoc™ (Bio-Rad). Densitometry was measured in ImageLab (version 5.2.1, build 11, Bio-Rad).

### Data quantification

Image analyses were performed in Fiji (ImageJ, version 2.0.0-rc-65/1:51u). GraphPad Prism 6 (La Jolla, CA, USA) was used for statistical analyses and to visualise the data. Previous data from our laboratory was used to determine sample size and data assumed to be normally distributed. Repeat numbers represent biological repeats. Randomly chosen N2A-FLAG-TrkB cells and ES-MNs were treated with BFA and BDNF. Datasets were analysed using unpaired two-tailed Student's *t*-test; χ*^2^* test was used to analyse the difference in endosome diameters in PTPN23-KD and scrambled cells. Unless stated otherwise, all graphs show mean values, and error bars show ±s.e.m.

## Supplementary Material

Supplementary information

Reviewer comments
