# Supplementary material for: PTPN23 binds the dynein adaptor BICD1 and is required for endocytic sorting of neurotrophin receptors
Source: J Cell Sci. 2020 Mar 30;133(6):jcs242412. doi: 10.1242/jcs.242412 (PMC7132798; doi:10.1242/jcs.242412)
Supplement: Supplementary information [file joces-133-242412-s1.pdf]

Supplementary Information

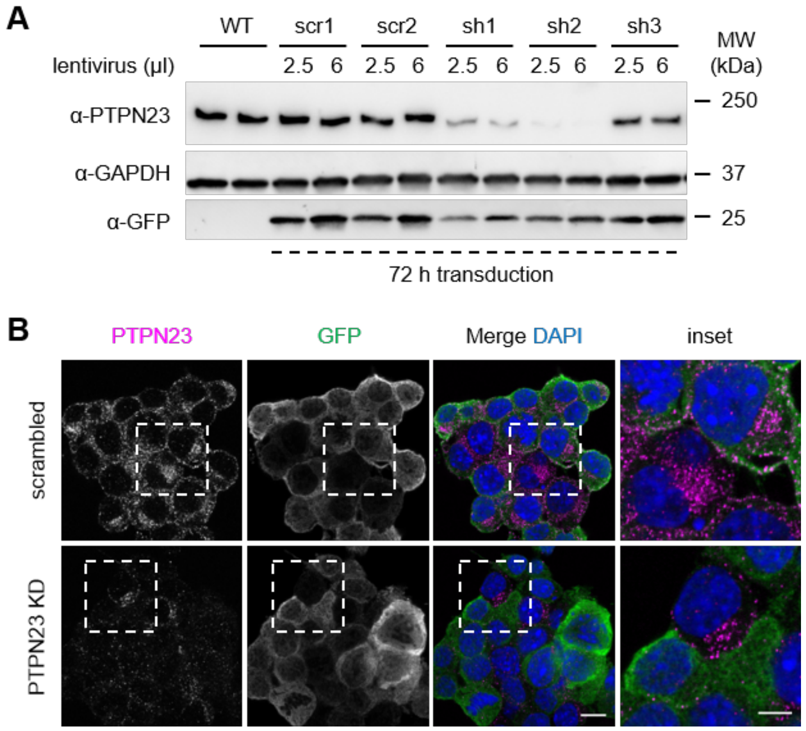

Fig. S1. PTPN23 is efficiently downregulated by sh1 and sh2 lentiviruses. (A) N2A-FLAG-TrkB cells were transduced with 2.5 or 6 µl of lentiviral suspension for 72 h (n=2). Cell lysates were immunoblotted for PTPN23 and GFP. GAPDH was used as a loading control. Sh2 lentivirus was the most efficient in silencing PTPN23, however, due to its toxicity, sh1 was chosen instead for further work. (B) Confocal images of N2A-FLAG-TrkB cells transduced for 72 h with PTPN23-targeting sh1 lentivirus or scrambled control, fixed and immunostained using anti-PTPN23 and anti-GFP antibodies (n=2). Scale bar: 10 µm; inset: 5 µm.

Journal of Cell Science • Supplementary information

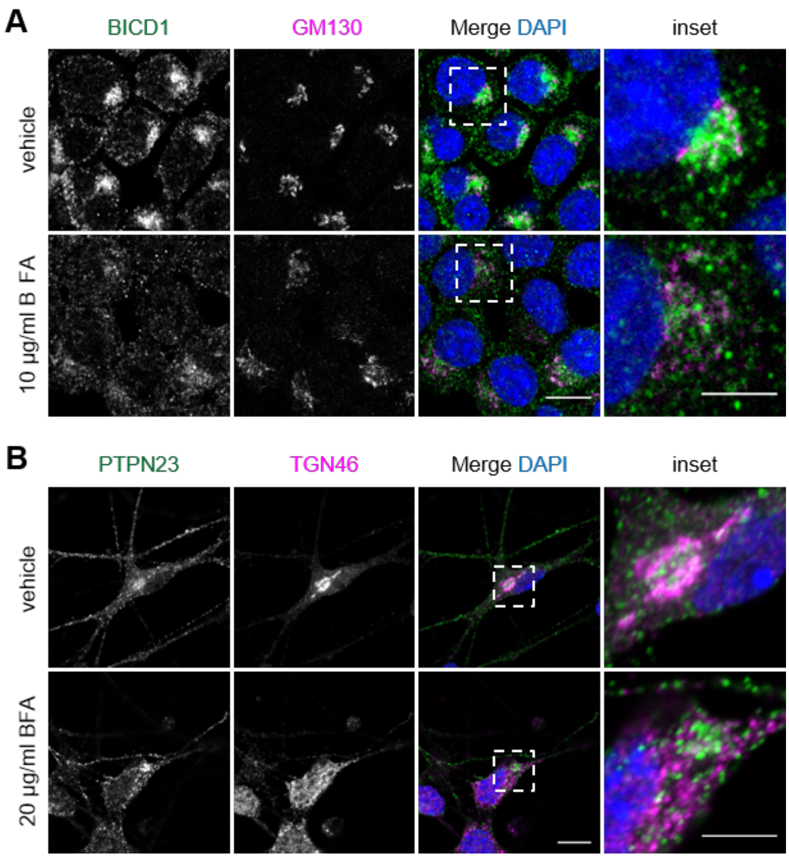

Fig. S2. The perinuclear enrichment of PTPN23 is independent of Golgi integrity. (A) Confocal images of N2A-FLAG-TrkB cells treated for 1 h with vehicle or 10 µg/ml brefeldin A (BFA), fixed and immunostained using anti-BICD1 and anti-GM130 antibodies (n=2). Scale bar: 10 µm; inset: 5 µm. (B) Confocal images of ES-MNs treated for 1 h with vehicle or 20 µg/ml brefeldin A (BFA), fixed and immunostained using anti-PTPN23 and anti-TGN46 antibodies (n=2). Scale bar: 10 µm; inset: 5 µm.

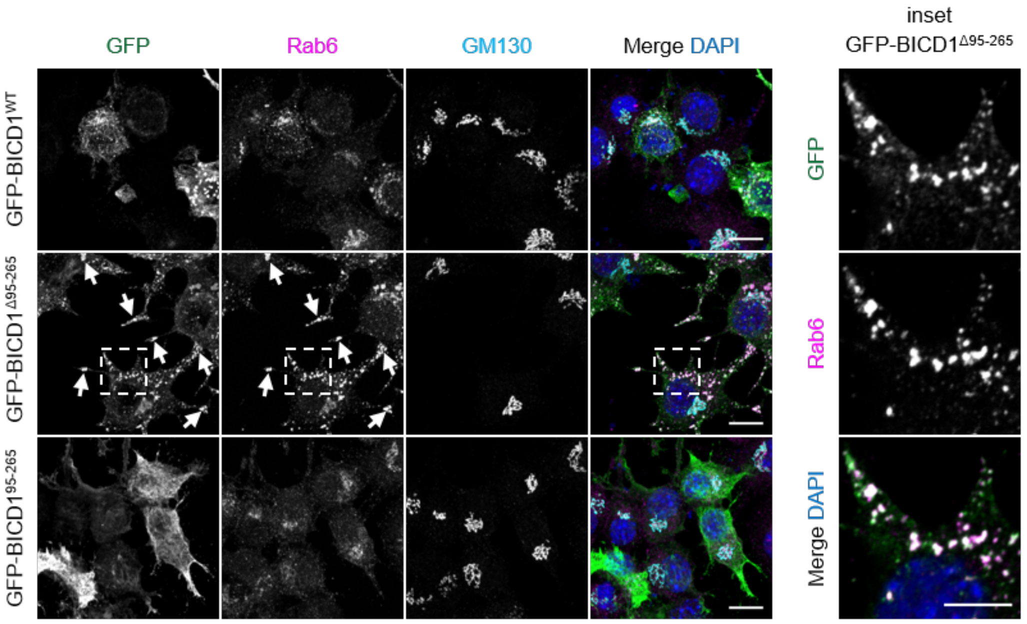

Fig. S3. GFP-BICD1$^{\Delta 95\text{-}265}$ induces peripheral redistribution of Rab6-positive vesicles. N2A-FLAG-TrkB cells, transfected overnight with different GFP-BICD1 proteins as indicated, were fixed and immunostained using anti-Rab6 and anti-GM130 antibodies (n=3). Rab6-positive vesicles translocate to the cell periphery with GFP-BICD1$^{\Delta 95\text{-}265}$ (arrows; see inset). Images show maximum intensity Z-stack projections, acquired at 0.5 µm spacing; insets show a representative frame selected from the respective Z-stack. Scale bar: 10 µm; inset: 5 µm.

Journal of Cell Science • Supplementary information

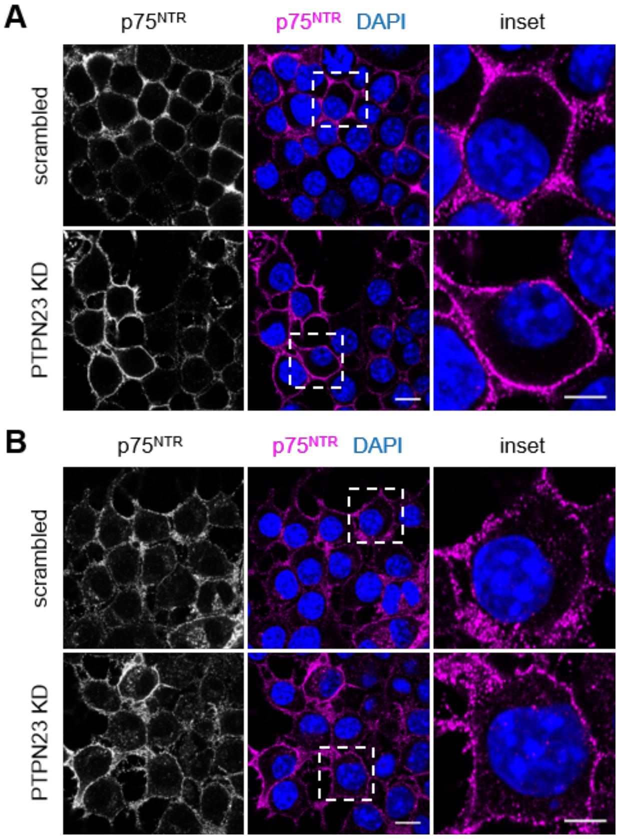

Fig. S4. PTPN23 is not required to maintain steady-state levels of p75^{NTR} at the plasma membrane. Confocal images showing surface (A; non-permeabilised cells) and total (B; permeabilised cells) immunostaining of p75^{NTR} in N2A-FLAG-TrkB cells, transduced with scrambled and PTPN23 shRNA specific lentiviruses (n=2). Scale bar: 10 μm; inset: 5 μm.

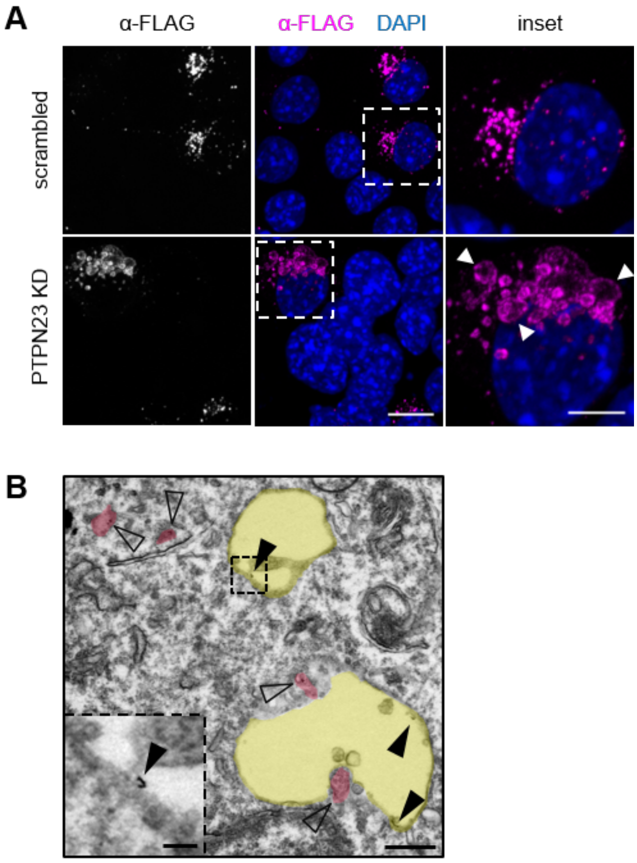

Fig. S5. Loss of PTPN23 leads to TrkB and p75^{NTR} accumulation in vacuole-like compartments. (A) Confocal images of α-FLAG(TrkB) accumulation in scrambled and PTPN23 shRNA treated N2A-FLAG-TrkB cells (n=3). Following acid-wash and fixation, receptor localisation was revealed using AlexaFluor555-conjugated secondary antibody. Arrowheads indicate enlarged endocytic compartments. Images show maximum intensity Z-stack projections, acquired at 0.5 μm spacing. Scale bar: 10 μm, inset: 5 μm. (B) Transmission electron microscopy images of PTPN23 shRNA treated N2A-FLAG-TrkB cells showing early endosomes (red) and an enlarged endocytic compartments (yellow; see inset), containing accumulated α-p75^{NTR}-gold (arrowheads). Scale bar: 500 nm; inset: 100 nm.

Journal of Cell Science • Supplementary information

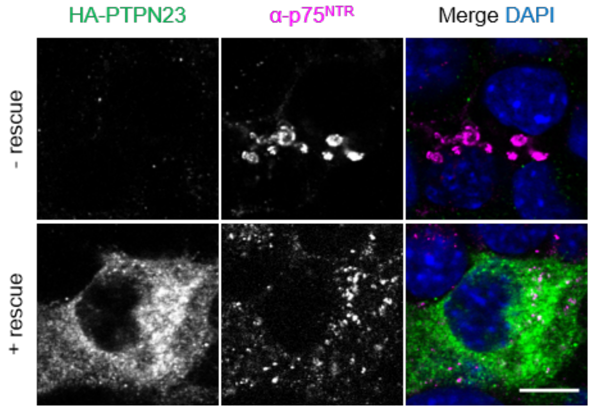

Fig. S6. HA-PTPN23 overexpression in PTPN23 KD cells rescues the α-p75^{NTR} accumulation phenotype. Confocal images of α-p75^{NTR} accumulation in PTPN23 KD cells, transfected overnight with shRNA-resistant HA-PTPN23 (n=2). Following antibody accumulation, N2A-FLAG-TrkB cells were acid-washed, fixed and immunostained using anti-HA antibody. Cells expressing HA-PTPN23 lack α-p75^{NTR} accumulation in vacuole-like compartments. Scale bar: 10 µm.

Table S1. BICD1 interactome. Complete list of the BICD1 interactors identified by mass spectrometry of BICD1 immunoprecipitates obtained from lysates of untreated and BDNF-stimulated ES-MNs and N2A-FLAG-TrkB cells. Common contaminants were identified by consulting the Contaminant Repository for Affinity Purification (Crapome; http://crapome.org/). BICD1 is highlighted in green, its close homologue BICD2 in light green, and PTPN23 in yellow.

Protein IDs:	Uniprot identifier associated with the identification.
Protein names:	Protein name extracted from Uniprot.
Gene names:	Mapped Gene name.
ES control log10 iBAQ:	Protein quantity as determined by MaxQuant using intensity based absolute quantification (iBAQ).
ES BDNF log10 iBAQ	Protein quantity as determined by MaxQuant using intensity based absolute quantification (iBAQ).
N2A control log10 iBAQ:	Protein quantity as determined by MaxQuant using intensity based absolute quantification (iBAQ).
N2A BDNF log10 iBAQ:	Protein quantity as determined by MaxQuant using intensity based absolute quantification (iBAQ).
Crapome frequency:	Frequency of occurrence in the Contaminant Repository for Affinity Purification Mass Spectrometry Data (Crapome) containing 411 experiments. A high number indicates a high likelihood of contaminant identification, a low number correlates with specificity.
GOBP name	Gene Ontology Biological Process terms.
GOMF name	Gene Ontology Metabolic Process.
GOCC name	Gene Ontology Cellular Compartment.

Signaling Endosome enriched (Debaisieux *et al*): Indicates proteins determined specific for the signalling endosome compartment according to the Debaisieux *et al.* (2016).

Q-value:	Q-value for protein identification, low values indicate confident identification. The estimated false discovery rate for the dataset is 1%.
MS/MS count:	Total number of spectra acquired for this protein.

Sequence coverage 15 min BDNF stimulated [%]: Sequence coverage 15 min BDNF stimulated [%].

Sequence coverage BDNF stimulated [%]: Sequence coverage BDNF stimulated [%].

Sequence coverage control [%]: Sequence coverage control [%].

Sequence coverage serum starved control [%]: Sequence coverage serum starved control [%].

Mol. weight [kDa]: Molecular weight [kDa].

Sequence length: Sequence length.

iBAQ: Intensity-Based Absolute Quantification.

NaN: Not a Number. No iBAQ value could be calculated for this protein in this condition.

Click here to Download Table S1

Table S2. Primers used for BICD1 and PTPN23 cloning

Target	Primer name	Sequence (5’ → 3’)
pET28a+	FW	TGAGATCCGGCTGCTAAC
	REV	CATATGGCTGCCGCG
pGEX-4T-1	FW	TGACTGACTGACGATCTGCC
	REV	GGATCCACGCGGAACC
pEGPFC1	FW	AACTGATCATAATCAGCCATACCAC
	Rev	AGCTTGAGCTCGAGATCTGAGTC
PTPN23 insert for pET28a+	BroFW8	**CGCGGCAGCCATATG**CCCATGATCTGGCTGGAC
	BroREV407	**TTAGCAGCCGGATCTCA**CGTCTCGGGATCCAACTG
	V/CCFW401	**CGCGGCAGCCATATG**ATGCAGTTGGATCCCGAG
	V/CCREV653	**TTAGCAGCCGGATCTCA**GGAGTTCCACTTTTGGTCC
	PR/HDFW703	**CCGCGCAGCCATATG**GCCCGCCAGCAGCTC
	PR/HDFW705	**CGCGGCAGCCATATG**CAGCAGCTCCTGGACAG
	PR/HDREV1134	**TTAGCAGCCGGATCTCA**AGGAGACTGAGTGCCGC
	PTPFW1188	**CCGCGCAGCCATATG**GTGGGAGCTCTGGACACTG
	PTPREV1452	**TTAGCAGCCGGATCTCA**GTGTCTCACCACTGCCTCATAG
	PTPREV1475	**TTAGCAGCCGGATCTCA**GCTGATGCTTGCACTGG
BICD1 insert for pGEX-4T-1	CC1FW1	**GTTCCGCGTGGATCC**ATGGCCGCAGAAGAGGTATTG
	CC1REV94	**GCAGATCGTCAGTCAGTCA**AAGCGTTTCCTCCCGAGT
	CC1FW95	**GTTCCGCGTGGATCC**CTGCAGGAGTCAGCATCG
	CC1REV265	**GCAGATCGTCAGTCAGTCA**GATGCTGATATGGTTATCATTGAG
	CC1REV300	**GCAGATCGTCAGTCAGTCA**ATAGTCTCCATTCAGTTTCACAAGAGG
	CC2FW314	**GTTCCGCGTGGATCC**GTCTCTGACTTATTCAGTGAGCTG
	CC2REV500	**GCAGATCGTCAGTCAGTCA**GGTACTGTGATTTTCGTTGGC
	CC3FW662	**GTTCCGCGTGGATCC**ATGATTGATAAAGACAAGGAAGCC
	CC3REV808	**GCAGATCGTCAGTCAGTCA**TTTGCTGCGTCGGGAC

Journal of Cell Science • Supplementary information

BICD1 insert for pEGFPC1	FWΔ265	GGAAGTGATATCGGAAGTTCAGTAGATGGACTCAAATTTGC
	REVΔ95	ACTTCCGATATCACTTCCAAGCGTTTCCTCCCGAGTC
	FW95	TCTCGAGCTCAAGCT**CTGCAGGAGTCAGCATCG**
	REV265	GATTATGATCAGTTA**GATGCTGATATGGTTATCATTGAG**

Journal of Cell Science • Supplementary information
