# Supplementary material for: PTPN23 binds the dynein adaptor BICD1 and is required for endocytic sorting of neurotrophin receptors
Source: J Cell Sci. 2020 Mar 30;133(6):jcs242412. doi: 10.1242/jcs.242412 (PMC7132798; doi:10.1242/jcs.242412)
Supplement: Reviewer comments [file joces-133-242412_review_history.pdf]

Journal of Cell Science

PTPN23 binds the dynein adaptor BICD1 and is required for endocytic sorting of neurotrophin receptors

Marta I. Budzinska, David Villarroel-Campos, Matthew Golding, Anne Weston, Lucy Collinson, Ambrosius P. Snijders and Giampietro Schiavo
DOI: 10.1242/jcs.242412

Editor: David Stephens

Review timeline

Original submission:	29 November 2019
Editorial decision:	10 January 2020
First revision received:	3 February 2020
Accepted:	4 February 2020

Original submission

First decision letter

MS ID#: JOCES/2019/242412

MS TITLE: PTPN23 is required for endocytic sorting of neurotrophin receptors via a BICD1-dependent mechanism

AUTHORS: Marta I Budzinska, David Villarroel-Campos, Matthew Golding, Ambrosius P Snijders, Anne Weston, Lucy Collinson, and Giampietro Schiavo
ARTICLE TYPE: Research Article

We have now reached a decision on the above manuscript.

To see the reviewers' reports and a copy of this decision letter, please go to: https://submit-jcs.biologists.org and click on the 'Manuscripts with Decisions' queue in the Author Area. (Corresponding author only has access to reviews.)

As you will see, the reviewers gave favourable reports but raised some critical points that will require amendments to your manuscript. I hope that you will be able to carry these out, because I would like to be able to accept your paper.

I agree with the key comment that the title is not fully supported by the data in that you do not link the interaction between BICD1 and PTPN23 to NTR trafficking directly. This should either be addressed by a rephrasing of the title or further experimentation. The latter would not be a prerequisite for acceptance. I also consider that you should show validation of your PTPN23 antibody labelling and provide some more details and better presentation of the mass spec data (while I appreciate that you have deposited the raw data, most readers will likely refer only to that within the manuscript itself).

Please ensure that you clearly highlight all changes made in the revised manuscript. Please avoid using 'Tracked changes' in Word files as these are lost in PDF conversion.

I should be grateful if you would also provide a point-by-point response detailing how you have dealt with the points raised by the reviewers in the 'Response to Reviewers' box. Please attend to

© 2020. Published by The Company of Biologists under the terms of the Creative Commons Attribution License (https://creativecommons.org/licenses/by/4.0/).

all of the reviewers' comments. If you do not agree with any of their criticisms or suggestions please explain clearly why this is so.

Reviewer 1

Advance summary and potential significance to field

This manuscript describes the interaction of the ESCRT interactor PTPN23 and the dynein regulator BICD1 and analyses their joint roles in the trafficking of neurotrophin receptors (e.g. p75NTR, TrkB) in neuronal cells. This is important given the role of signalling endosomes in controlling neuronal cell survival and function.
PTPN23 has already been broadly established as a regulator of endosomal trafficking of several receptors but so far not NTRs. This manuscript is particularly important because it identifies a defect (albeit not fully penetrant) in sorting of NTRs to the lumen of endosomes. It also is the first to report a direct link between the endosomal sorting machinery and microtubule motors, a finding that will have broad impact given that endosomal sorting and movement are most likely mechanistically linked.

The interaction data are convincing, despite the relative weakness (and presumably transient nature) of the interaction between PTPN23 and BICD1, and likewise the interaction mapping studies are robust. The effects of PTPN23 reduction on NTR trafficking are clear, and in line with the effects on other receptors. However, I believe the manuscript could be strengthened by changes.

Comments for the author

1. Reconfigure Table S1. It’s not clear from this table why PTPN23 was chosen as one of the highest scoring hits, nor the impact of BDNF on the interaction. Was PTPN23 chosen because the interaction altered upon NTR activation? Or simply because of its likely functional relevance? Give more information about the screen conditions, methodology used to threshold and select for candidate hierarchy etc.
2. Conduct control experiments to identify whether the PTPN23 staining is real. For example, perform a kd of PTPN23.
3. Page 10. It is over-interpreting to say that BICD1 delta95-265 exerts dominant-negative effects based solely on a single IF experiment. No function is measured. In all, I’m not convinced that the experiments examining Rab6 localisation tell us anything, especially given that the deletion mutant is not able to bind dynein/dynactin. The loss of this interaction, rather than that with PTPN23, is more likely to explain the localisation defect reported here.
4. Figure 6D. It is important to identify which example panels are from control cells and which from KD cells. Indeed, the authors should include examples from both control and KD cells to highlight the morphological differences shown in the accompanying bar chart.
5. It would enhance the interest in this report if the authors could speculate in the Discussion about the potential links between the defects in neurotrophin receptor trafficking they observe and the reported neurological defects attributed to PTPN23 mutation. For example, is it possible that defective receptor sorting underlies the observed defects in neuronal pruning?

Reviewer 2

Advance summary and potential significance to field

The authors present interesting work looking at the interaction partners of BICD1 and how this may relate to the role of BICD1 in sorting and trafficking of NTRs.
Following mass spec analysis, the authors choose to follow up on PTPN23, an ESCRT protein with known roles in receptor trafficking. The interaction is well characterised; Figure 1 shows endogenous IP of BICD1 with PTPN23, but relatively poor overlap of endogenous components by immunofluorescence. Consequently Figure 2 is important as it shows detailed in vitro protein-protein interactions confirming the interaction. Following on from characterising the interaction, the authors demonstrate a clear phenotype of PTPN23 knockdown on NTR trafficking in N2A cells. Figure 4, 5, 6 and 7 use PTPN23 knockdown in N2A cells to show that PTPN23 has a role in sorting

© 2020. Published by The Company of Biologists under the terms of the Creative Commons Attribution License (https://creativecommons.org/licenses/by/4.0/).

NTRs, where knockdown causes accumulation of NTRs in sorting endosomes that form enlarged vacuoles. This phenotype is rescued by overexpression (Supplementary Figure 5). A similar phenotype has already been observed for BICD1 depletion in ES-MNs (Terenzio, 2014). However a direct link between the BICD1-PTPN23 interaction and NTR sorting - the title of the paper - has not been made experimentally.

Comments for the author

Major issues

1. As presented there is currently insufficient evidence to support the title of the paper - the interaction between BICD1 and PTPN23 is not linked to NTR trafficking.
Unless this can be resolved experimentally, then it's unclear how the first half of the paper relates to the second half. Can the vacuole phenotype with PTPN23 KD be rescued with BICD1 overexpression or visa versa? Alternatively, what effect does the overexpression of BICD1-CC1 have on NTR trafficking and is it the same as PTPN23-V/CC? Please can the authors supply some experimental evidence that supports the link in their title.

2. There is currently no presentation of mass spec results and also no indication of how many biological repeats were carried out. How similar were hits from N2A and ES cells? Did any of the hits show enrichment with BDNF treatment? In addition Supplementary Table 1 can't be read - for example where multiple gene IDs are present in the first column, only the first can be seen. Please include as an .xlsx file.

3. Figure 3 seems at odds with the main thrust of the paper. The Rab6 result in D should be pushed to the supplement as it seems unrelated to the main findings. A and B could easily be incorporated into Figure 2. Overexpression IF in C is unconvincing and this assay needs some quantification to be meaningful. I would consider leaving this out, given that the authors are very clear that there is little overlap in the first figures looking at endogenous proteins. The biochemistry proves the interaction sufficiently.

Minor issues
1. I appreciate the grey panels for single channel images, but the use of colour is problematic. Swap red for magenta to make two colour cell images accessible to colourblind readers. The best three colour overlay is magenta, yellow and cyan. In this case I would consider removing the DAPI channel (Figure 3D).

First revision

Author response to reviewers' comments

Professor David Stephens
Editor, Journal of Cell Science

Re: JOCES/2019/242412

Dear David,
Thank you for your time and efforts in handling our manuscript JOCES/2019/242412 entitled "PTPN23 is required for endocytic sorting of neurotrophin receptors via a BICD1-dependent mechanism". We appreciated your editorial comments, and the encouragement and constructive critique provided by the two Reviewers, which pinpointed to a few shortcomings and minor inaccuracies in the interpretation of our results.

We have now revised the manuscript in light of these comments. As a result, this amended version is vastly improved, thus meeting the strict quality requirement of JCS. Please find below a point-by-point response to these comments:

© 2020. Published by The Company of Biologists under the terms of the Creative Commons Attribution License (https://creativecommons.org/licenses/by/4.0/).

<u>Editorial Comments</u>
A. I agree with the key comment that the title is not fully supported by the data in that you do not link the interaction between BICD1 and PTPN23 to NTR trafficking directly. This should either be addressed by a rephrasing of the title or further experimentation. The latter would not be a prerequisite for acceptance.

Thank you for this comment. We have rephrased the title as “PTPN23 binds the dynein adaptor BICD1 and is required for endocytic sorting of neurotrophin receptors.” We believe that this title better represents our findings and should avoid data overinterpretation.

B. I also consider that you should show validation of your PTPN23 antibody labelling.

To address this important point, we have performed shRNA knockdown of PTPN23 in N2A-FLAG-TrkB cells and then stained them, together with N2A cells treated with shRNA scrambled control, with the anti-PTPN23 antibody used in Fig. 1. As show in Fig. S1, we were able to almost completely abolish the PTPN23 signal both in western blot (Fig. S1A) and immunofluorescence (Fig. S1B), thus demonstrating the specificity of this antibody and the validity of our conclusions.

We apologise with the Editor and Reviewer 1 that these conclusions were not sufficiently highlighted in the first version of our submitted manuscript, and that the panel showing these results was not optimal. We have now amended the main text on page 6 and changed Fig. S2B (now Fig. S1B).

C. and provide some more details and better presentation of the mass spec data (while I appreciate that you have deposited the raw data, most readers will likely refer only to that within the manuscript itself).

We have discussed with our mass-spec collaborators about a better representation of the mass specrometry data, which would maximise their readability and access to a wider audience. To this end, we have added to the main text a simplified version of Table S1 containing only proteins associated to BICD1 immunoprecipitates, which have been previously involved in intracellular transport and localisation (Table 1; GO terms listed in the legend), and found associated with BICD1 beads upon incubation with both N2A and ES-derived motor neuron lysates with or without BDNF stimulation.

<u>Reviewer 1</u>
1. Reconfigure Table S1. It’s not clear from this table why PTPN23 was chosen as one of the highest scoring hits, nor the impact of BDNF on the interaction. Was PTPN23 chosen because the interaction altered upon NTR activation? Or simply because of its likely functional relevance? Give more information about the screen conditions, methodology used to threshold and select for candidate hierarchy etc.

As stated above (reply to editorial comment C), we have added a simplified version of Table S1 to the main text. In addition, we have modified the manuscript on page 5 to include the criteria for selection of PTPN23 among the hits listed in Table 1 and S1. These criteria were the presence of PTPN23 in BICD1 immunoprecipitates obtained both from N2A and ES-derived motor neuron lysates in BDNF stimulated and unstimulated conditions, its established role in membrane traffic and the specific phenotype induced by its downregulation (formation of large vacuoles), which is morphologically similar to that observed in BICD1 knockout cells. Furthermore, we omitted from our analysis protein commonly found associated to control beads (https://www.crapome.org). See also point 2 of Reviewer 2.

2. Conduct control experiments to identify whether the PTPN23 staining is real. For example, perform a knockdown of PTPN23.

Please see the answer to point B raised by the Editor.
3. Page 10. It is over-interpreting to say that BICD1 delta95-265 exerts dominant-negative effects based solely on a single IF experiment. No function is measured. In all, I’m not convinced that the experiments examining Rab6 localisation tell us anything, especially given that the deletion mutant

© 2020. Published by The Company of Biologists under the terms of the Creative Commons Attribution License (https://creativecommons.org/licenses/by/4.0/).

is not able to bind dynein/dynactin. The loss of this interaction, rather than that with PTPN23, is more likely to explain the localisation defect reported here.

Following the reviewer's suggestion, we have tuned down the emphasis on the possible dominant-negative behaviour of GFP-BICD1 95-265 (page 8) and shifted Fig. 3D, which is focussed on the effects of BiCD1 mutants on Rab6 localisation, to the supplementary section (new Fig. S3). See also point 3 of Reviewer 2.

4. Figure 6D. It is important to identify which example panels are from control cells and which from KD cells. Indeed, the authors should include examples from both control and KD cells to highlight the morphological differences shown in the accompanying bar chart.

The source of the example panels shown in Fig. 6D have now been identified in the corresponding legend. Whilst the morphology of tubulo-vesicular organelles/early endosomes and late endosomes/lysosomes are indistinguishable in scrambled control and PTPN23 knockdown samples, large vacuoles are only present upon PTPN23 downregulation.

5.It would enhance the interest in this report if the authors could speculate in the Discussion about the potential links between the defects in neurotrophin receptor trafficking they observe and the reported neurological defects attributed to PTPN23 mutation. For example, is it possible that defective receptor sorting underlies the observed defects in neuronal pruning?

We thank the Reviewer for this comment. We have changed the Discussion as suggested.

Reviewer 2

1. As presented, there is currently insufficient evidence to support the title of the paper - the interaction between BICD1 and PTPN23 is not linked to NTR trafficking. Unless this can be resolved experimentally, then it's unclear how the first half of the paper relates to the second half. Can the vacuole phenotype with PTPN23 KD be rescued with BICD1 overexpression or visa versa? Alternatively, what effect does the overexpression of BICD1-CC1 have on NTR trafficking and is it the same as PTPN23-V/CC? Please can the authors supply some experimental evidence that supports the link in their title.

Some of the suggested experiments have been attempted but due to a series of technical shortcomings, these results were not reliable and therefore they have not been added to the manuscript. In particular, overexpressed PTPN23-V/CC accumulated in large, heavily clustered vacuoles/aggregates, which are not enriched in endocytic markers but are positive for ubiquitin. TrkB dynamics appeared not to be affected in these cells, however this experiment was done in the background of endogenous PTPN23 and BICD1. Likewise, TrkB/p75 internalization was attempted in cells overexpressing GFP-BICD1D95-265, but not BICD1-CC1 in the presence of endogenous levels of PTPN23 and BICD1, and no major effects of this mutant on NTR accumulation were observed. The high basal level of AKT and ERK1/2 activation in N2A cells precluded us to carry out signalling experiments with BDNF.

2. There is currently no presentation of mass spec results and also no indication of how many biological repeats were carried out. How similar were hits from N2A and ES cells? Did any of the hits show enrichment with BDNF treatment? In addition, Supplementary Table 1 can't be read - for example where multiple gene IDs are present in the first column, only the first can be seen. Please include as an .xlsx file.

We apologise with this Reviewer for the lack of clarity in describing the results displayed in Table S1, and for the mis-formatting caused by the conversion of the original Excel (.xlsl) format to pdf, which caused problems with the full access to the data. As described in our response to point 1 of the first Reviewer and to the Editor, the most relevant binding partners of BICD1 have now been added to the main text as Table 1. Furthermore, Table S1 has now been uploaded in .xlsl format, which would allow full access to all gene IDs found.

The mass-spec data were obtained by BICD1 immunoprecipitates obtained in two independent experiments using N2A and ES-derived motor neuron lysates with or without BDNF stimulation. The abundance of a certain protein in different samples is shown in columns D-G of Table S1, and the

© 2020. Published by The Company of Biologists under the terms of the Creative Commons Attribution License (https://creativecommons.org/licenses/by/4.0/).

relative enrichment of a certain protein in different samples might be inferred by the comparison of the iBAQ values.

3. Figure 3 seems at odds with the main thrust of the paper. The Rab6 result in D should be pushed to the supplement as it seems unrelated to the main findings. A and B could easily be incorporated into Figure 2. Overexpression IF in C is unconvincing and this assay needs some quantification to be meaningful. I would consider leaving this out, given that the authors are very clear that there is little overlap in the first figures looking at endogenous proteins. The biochemistry proves the interaction sufficiently.

Following the reviewer's suggestion, we have shifted Fig. 3D to the supplementary section (new Fig. S3), and tuned down the emphasis on the possible dominant-negative behaviour (page 8-9). However, we would prefer to leave Fig. 2 and the remaining panels of Fig. 3 separated, since we believe that this arrangement increases the readability of the data.

Minor issues: 1. I appreciate the grey panels for single channel images, but the use of colour is problematic. Swap red for magenta to make two colour cell images accessible to colour-blind readers. The best three colour overlay is magenta, yellow and cyan. In this case I would consider removing the DAPI channel (Figure 3D).

Thank you for the comment. Whilst we always try to avoid the use of red/green for binary images and using single colour panels to improve readability, it is difficult to choose a suitable palette in case of multicolour images. Following the comment of this reviewer, we have replaced red for magenta throughout the manuscript.
However, in the case of Figure 3D, dropping the DAPI staining would make the figure more difficult to interpret, henceforth we respectfully ask to keep DAPI in the images.

<u>Second decision letter</u>

MS ID#: JOCES/2019/242412

MS TITLE: PTPN23 binds the dynein adaptor BICD1 and is required for endocytic sorting of neurotrophin receptors

AUTHORS: Marta I Budzinska, David Villarroel-Campos, Matthew Golding, Anne Weston, Lucy Collinson, Ambrosius P Snijders, and Giampietro Schiavo
ARTICLE TYPE: Research Article

Thank you for the careful revisions to your manuscript. I am happy to tell you that your manuscript has been accepted for publication in Journal of Cell Science, pending standard ethics checks.

© 2020. Published by The Company of Biologists under the terms of the Creative Commons Attribution License (https://creativecommons.org/licenses/by/4.0/).
